# Optimization of solid-state fermentation conditions for high β-galactosidase-producing lactic acid bacteria and its application in low-lactose dairy products

**DOI:** 10.3389/fbioe.2025.1708601

**Published:** 2025-12-11

**Authors:** Zhanjia Zhang

**Affiliations:** Food Science, College of Food, Agricultural and Natural Resource Sciences, University of Minnesota, St Paul, MN, United States

**Keywords:** β-galactosidase, *Lactobacillus* plantarum, solid-state fermentation, low-lactose dairy products, response surface methodology

## Abstract

**Introduction:**

Lactose intolerance affects 85%–95% of Chinese adults, creating substantial demand for low-lactose dairy products. This study aimed to develop a cost-effective β-galactosidase production process through solid-state fermentation (SSF) using agricultural byproducts.

**Methods:**

A high-yielding strain was isolated from Tibetan fermented yak milk and identified through morphological, biochemical, and 16S rDNA sequence analysis. Solid-state fermentation conditions were optimized using single-factor experiments and Box-Behnken response surface methodology. Enzymatic properties were characterized, and the enzyme was applied to milk lactose hydrolysis. Techno-economic and environmental impact analyses were conducted.

**Results:**

Lactobacillus plantarum LP-15 exhibited initial enzyme activity of 44.7 U/g. Optimal SSF conditions were determined as substrate ratio of wheat bran:soybean meal:whey powder (6:3:1), 37 °C, pH 6.5, and 55% moisture content, achieving enzyme activity of 186.3 U/g (4.17-fold improvement). The enzyme exhibited optimal activity at pH 6.5 and 42 °C, with Km of 2.8 mM and catalytic efficiency of 5.3 × 10^4^ M^−1^s^−1^. Milk lactose was reduced by 81.9% within 4 h using 2.0 U/mL enzyme at 40 °C, meeting the low-lactose standard (≤0.1%). SSF reduced production costs by 35.7%, water usage by 94%, and CO_2_ emissions by 62.4% compared to liquid fermentation.

**Discussion:**

This study provides an economically viable and environmentally sustainable solution for low-lactose dairy production in China, establishing independent intellectual property rights while addressing nutritional needs of lactose-intolerant populations through circular utilization of agricultural by products.

## 1 Introduction

Lactose intolerance results from reduced or absent lactase activity in the small intestinal mucosa, leading to incomplete lactose hydrolysis and subsequent fermentation in the intestine, producing gas and organic acids that cause gastrointestinal symptoms such as bloating and diarrhea (Facioni et al., 2020). Statistics indicate that approximately 65% of adults worldwide experience varying degrees of lactose malabsorption, with prevalence rates reaching 70%–100% in Asian populations and 85%–95% among Chinese adults (Jiao et al., 2025; Yang et al., 2023). However, with improving living standards, dairy products have become an essential component of modern diets due to their rich content of high-quality proteins and calcium. This contradiction between nutritional requirements and physiological limitations has created a substantial market for low-lactose dairy products (Zheng et al., 2023). In 2024, the global low-lactose/lactose-free dairy market reached $18.5 billion, with the Chinese market valued at approximately 120 billion RMB and growing rapidly (China Dairy Association, 2024).

β-Galactosidase (EC 3.2.1.23) is the key enzyme catalyzing lactose hydrolysis (Ruiz-Ramírez and Jiménez-Flores, 2023). Currently commercialized β-galactosidases are primarily derived from yeasts and fungi, such as Novozymes' Lactozym and DSM’s Maxilact. While these products are technologically mature, they present challenges including high costs and import dependency. Lactic acid bacteria, particularly *Lactobacillus plantarum*, as GRAS-certified probiotics, produce β-galactosidase with optimal activity at pH 6.0–7.0 and 35 °C–45 °C, conditions that match milk processing requirements and show weak product inhibition (Delgado-Fernandez et al., 2020; Huang et al., 2025; Binda et al., 2020). However, wild strains exhibit low enzyme production levels (20–50 U/g), necessitating strain screening and process optimization to enhance enzyme production capacity (Kolev et al., 2021; Mahadevaiah et al., 2020).

Traditional liquid fermentation presents challenges including high medium costs, substantial energy consumption, and excessive wastewater generation. Solid-state fermentation (SSF) technology can directly utilize agricultural byproducts such as wheat bran and soybean meal, reducing raw material costs by 60%–80%, water usage and wastewater by over 70%, and in some cases achieving higher enzyme yields (Chilakamarry et al., 2022; Mattedi et al., 2023; Yafetto, 2022). Internationally, countries like India and Brazil lead in SSF technology applications, achieving production costs only 30%–40% of liquid fermentation. China possesses abundant agricultural byproduct resources and a tradition of solid-state fermentation, offering unique advantages for applying SSF technology to β-galactosidase production (Su et al., 2021; Kumar et al., 2021). However, lactic acid bacteria have high nutritional requirements, necessitating precise optimization of their solid-state fermentation conditions.

Based on these considerations, this study aims to screen high β-galactosidase-producing lactic acid bacteria from traditional fermented dairy products, establish an agricultural byproduct-based solid-state fermentation process, and develop low-cost enzyme production technology with independent intellectual property rights. The research employs single-factor experiments and Box-Behnken response surface methodology to optimize fermentation conditions (Liu et al., 2025; Menhart et al., 2020), systematically characterize enzymatic properties, and apply them to low-lactose dairy product development (Schulz and Rizvi, 2023). Industrial feasibility is evaluated through techno-economic analysis (Artola et al., 2024; Balina et al., 2023; Bradford, 1976).

This research holds significant importance: technically, it overcomes liquid fermentation limitations and establishes independent intellectual property rights; economically, it reduces costs by over 35% through agricultural byproduct utilization; socially, it addresses nutritional issues for lactose-intolerant populations and improves public health; environmentally, it achieves resource utilization of agricultural byproducts, aligning with green development principles. The study employs a technical route of “strain screening → fermentation optimization → enzymatic characterization → product application → economic analysis,” forming a complete technical chain to provide key technical support for China’s low-lactose dairy industry development.

## 2 Materials and methods

### 2.1 Materials

#### 2.1.1 Strain sources

Experimental samples were collected from 10 traditional fermented dairy products from Inner Mongolia, Xinjiang, Tibet, and Yunnan, including kumiss, cheese, fermented yak milk, and rushan. Samples were transported in ice boxes at 4 °C and subjected to strain isolation within 24 h. The standard strain *Lactobacillus plantarum* ATCC 8014 was purchased from the China Center of Industrial Culture Collection (CICC) and used as a control strain.

#### 2.1.2 Culture media

MRS medium (g/L): peptone 10.0, beef extract 10.0, yeast extract 5.0, glucose 20.0, Tween-80 1.0 mL, dipotassium hydrogen phosphate 2.0, sodium acetate 5.0, diammonium citrate 2.0, magnesium sulfate 0.58, manganese sulfate 0.25, agar 15.0 (for solid medium), pH 6.5 ± 0.2.

Screening medium (g/L): Based on MRS medium with lactose 20.0 replacing glucose, supplemented with X-gal (5-bromo-4-chloro-3-indolyl-β-D-galactopyranoside) 0.06 for initial screening of β-galactosidase-producing strains (Kolev et al., 2021).

Seed medium: MRS liquid medium supplemented with lactose 10.0 g/L as inducer.

Solid-state fermentation substrates: Wheat bran (Hebei Shijiazhuang Flour Factory), soybean meal (Shandong Rizhao Soybean Processing Factory), whey powder (Inner Mongolia Yili Dairy), all passed through 40-mesh sieve and dried at 105 °C to constant weight (Su et al., 2021).

#### 2.1.3 Main reagents

o-Nitrophenyl-β-D-galactopyranoside (ONPG) and o-nitrophenol (ONP) were purchased from Sigma-Aldrich; lactose, glucose, and galactose standards from Shanghai Aladdin Biochemical Technology; glucose assay kit from Nanjing Jiancheng Bioengineering Institute; bacterial genomic DNA extraction kit from Tiangen Biotech; PCR-related reagents from Takara Bio (Dalian); all other reagents were of analytical grade from domestic suppliers.

#### 2.1.4 Main instruments and equipment

UV-visible spectrophotometer (UV-2550, Shimadzu, Japan); High-performance liquid chromatography system (Waters e2695, Waters, United States); Thermal cycler (T100, Bio-Rad, United States); Gel imaging system (Gel Doc XR+, Bio-Rad, United States); Constant temperature incubator (SPX-250B, Shanghai Boxun); Constant temperature shaker (THZ-100, Shanghai Yiheng); High-speed refrigerated centrifuge (Avanti J-26XP, Beckman Coulter, United States); pH meter (FE28, Mettler-Toledo, Switzerland); Electronic balance (ML204, Mettler-Toledo, Switzerland).

### 2.2 Methods

#### 2.2.1 Isolation and screening of high β-galactosidase-Producing strains

**FIGURE 1:**
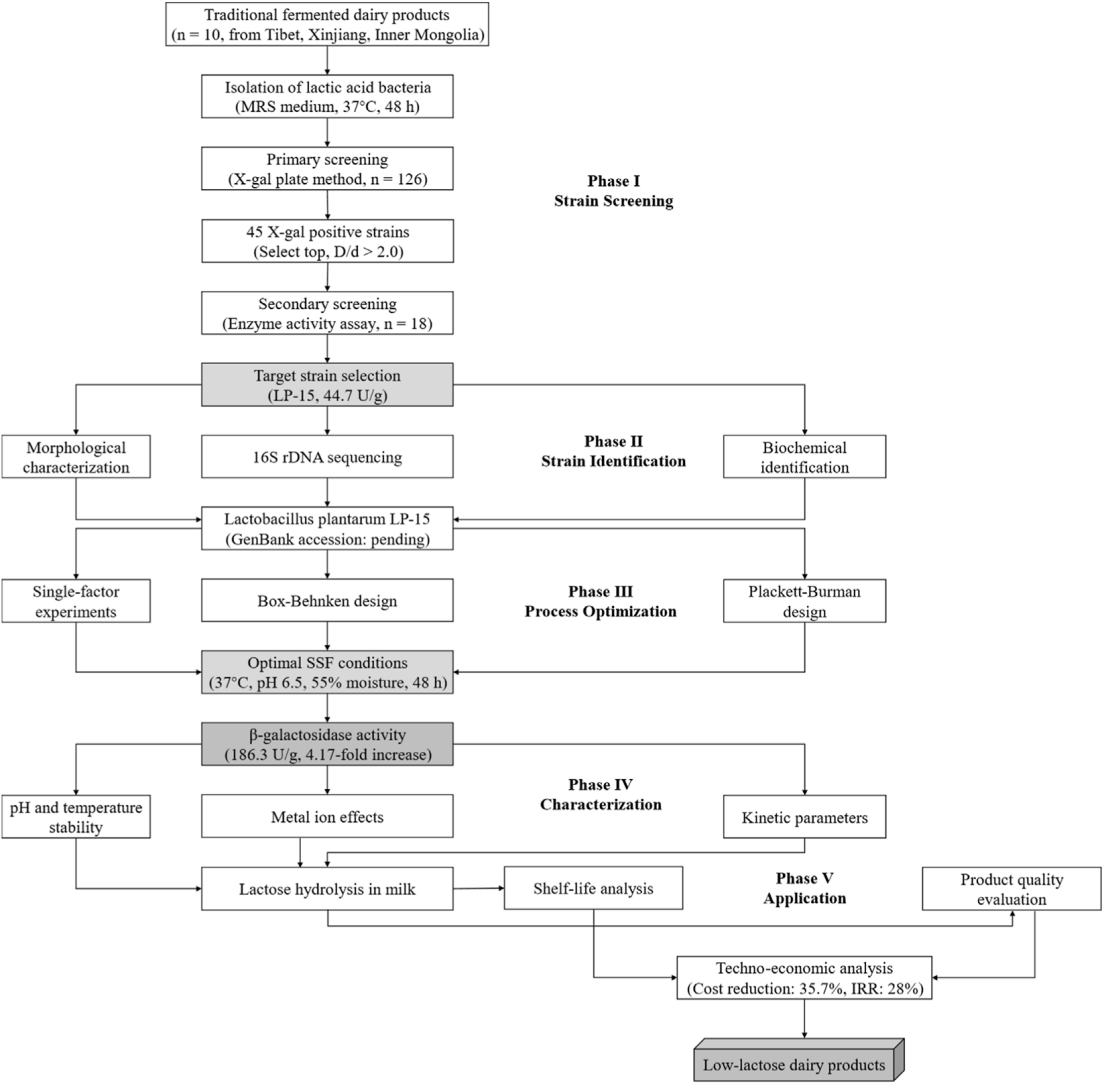
Technical route for screening of high β-galactosidase-producing lactic acid bacteria, optimization of solid-state fermentation, and application in low-lactose dairy products.

The overall technical route of this study is shown in Figure 1, employing a systematic research strategy of “strain screening → fermentation optimization → enzymatic characterization → product application → economic analysis.” The specific methods for each step are as follows.

Strain Isolation: 10 g of fermented dairy product samples were added to 90 mL sterile saline, mixed by shaking for 30 min, and serially diluted to 10^−6^. 100 μL of 10^−4^, 10^−5^, and 10^−6^ dilutions were spread on MRS solid medium plates and incubated anaerobically at 37 °C for 48 h. Single colonies with different morphologies were picked, purified three times, and preserved.

Primary Screening: Purified strains were point-inoculated on screening medium plates containing X-gal and incubated at 37 °C for 24 h. β-Galactosidase-producing strains hydrolyzed X-gal to produce blue color. The ratio of blue zone diameter (D) to colony diameter (d) was measured, and strains with D/d > 2.0 were selected for secondary screening (Kolev et al., 2021; Mahadevaiah et al., 2020).

Secondary Screening: From the 45 strains identified in primary screening, 18 strains with the highest D/d ratios (>2.0) were selected for secondary screening. These strains were inoculated in seed medium and cultured at 37 °C for 24 h, then transferred to MRS liquid medium (containing 20 g/L lactose) at 5% inoculation rate and cultured at 37 °C for 48 h. Cells were collected by centrifugation, disrupted by sonication, and crude enzyme β-galactosidase activity was determined. The strain with highest enzyme activity was selected for subsequent studies.

Strain Identification: High-producing strains were identified through morphological observation (Gram staining, scanning electron microscopy), physiological and biochemical identification (sugar fermentation test, gas production test, salt tolerance, etc.), and 16S rDNA sequence analysis. Universal primers 27F (5′-AGA​GTT​TGA​TCC​TGG​CTC​AG-3′) and 1492R (5′-GGT​TAC​CTT​GTT​ACG​ACT​T-3′) were used to amplify 16S rDNA. PCR products were sequenced by Sangon Biotech (Shanghai). Sequences were submitted to NCBI for BLAST comparison, and phylogenetic trees were constructed using MEGA 7.0 software.

#### 2.2.2 β-Galactosidase activity assay

Enzyme activity was determined using the ONPG method (Ruiz-Ramírez and Jiménez-Flores, 2023; Delgado-Fernandez et al., 2020). Reaction system: 0.1 mL appropriately diluted enzyme solution, 0.9 mL phosphate buffer (50 mM, pH 6.5) containing 5 mM ONPG, incubated at 37 °C for 10 min, reaction terminated by adding 1 mL of 1 M Na_2_CO_3_. Absorbance was measured at 420 nm, and ONP production was calculated using an ONP standard curve.

Enzyme activity unit (U) definition: Under the above conditions, the amount of enzyme required to hydrolyze ONPG and produce 1 μmol ONP per minute is defined as one enzyme activity unit. Solid-state fermentation enzyme activity is expressed as U/g dry substrate.

##### 2.2.2.1 Protein concentration determination

Protein concentration in crude enzyme extracts was determined using the Bradford assay (Taiwo et al., 2023). Briefly, 100 μL of appropriately diluted enzyme solution was mixed with 3 mL of Bradford reagent (Sangon Biotech, Shanghai, China). After incubation at room temperature for 5 min, absorbance was measured at 595 nm using a UV-visible spectrophotometer (UV-2550, Shimadzu, Japan). Protein concentration was calculated using a standard curve prepared with bovine serum albumin (BSA) standards ranging from 0.1 to 1.0 mg/mL. All measurements were performed in triplicate.

Specific activity (U/mg protein) was calculated as the ratio of enzyme activity (U) to total protein content (mg) in the crude enzyme extract. Both volumetric activity (U/g dry substrate) and specific activity (U/mg protein) were used to comprehensively evaluate enzyme production: the former reflects overall SSF process efficiency and practical application potential, while the latter indicates the intrinsic catalytic capacity of the enzyme and facilitates comparison with literature values.

#### 2.2.3 Optimization of solid-state fermentation conditions

Single-Factor Experimental Design: All single-factor experiments were conducted with the following baseline conditions unless otherwise specified: 10 g total substrate, 50% (w/w) moisture content, 37 °C incubation temperature, initial pH 6.5 (adjusted with sterile water), 10% (v/w) inoculum size (seed culture at OD600 = 1.8 ± 0.1), and 48 h fermentation time. Each factor was optimized sequentially while maintaining other parameters at baseline or previously optimized levels.

Solid-state fermentation was conducted in 250-mL Erlenmeyer flasks containing 10 g of mixed substrate (dry weight basis) with a layer thickness of approximately 1.5–2.0 cm. Substrates were sterilized by autoclaving at 121 °C for 15 min before moisture adjustment and inoculation. Initial moisture content was adjusted by adding calculated amounts of sterile distilled water. During fermentation, moisture loss was monitored by weighing flasks every 12 h, and sterile water was added as needed to maintain the target moisture content within ±2%. Flask openings were covered with cotton plugs wrapped in aluminum foil to allow gas exchange while minimizing evaporation. Flasks were incubated with relative humidity maintained at ≥85% by placing open containers of sterile water in the incubator. The substrate bed was manually mixed by gentle shaking every 24 h under aseptic conditions to ensure uniform oxygen distribution.

##### 2.2.3.1 Single-factor experiments

1. Substrate ratio: With 10 g total substrate, wheat bran: soybean meal: whey powder ratios of 10:0:0, 8:2:0, 6:4:0, 6:3:1, and 5:3:2 were tested. Fixed conditions: 50% moisture, 37 °C, pH 6.5, 10% inoculum, 48 h fermentation (Su et al., 2021).
2. Moisture content: Using the optimal substrate ratio (6:3:1) from experiment 1, moisture contents of 45%, 50%, 55%, 60%, and 65% (w/w) were tested. Fixed conditions: 37 °C, pH 6.5, 10% inoculum, 48 h (Kumar et al., 2021).
3. Fermentation temperature: Using optimal substrate ratio (6:3:1) and moisture (55%), temperatures of 30 °C, 33 °C, 37 °C, 40 °C, and 43 °C were tested. Fixed conditions: pH 6.5, 10% inoculum, 48 h.
4. Initial pH: Using optimized conditions (6:3:1 substrate, 55% moisture, 37 °C), pH was adjusted to 5.0, 5.5, 6.0, 6.5, 7.0, 7.5, and 8.0 using 0.1 M lactic acid or 0.1 M NaOH. Fixed conditions: 10% inoculum, 48 h.
5. Inoculum size: Using optimized conditions (6:3:1 substrate, 55% moisture, 37 °C, pH 6.5), inoculum sizes of 5%, 7.5%, 10%, 12.5%, and 15% (v/w) were tested with seed culture at OD600 = 1.8 ± 0.1. Fermentation time: 48 h.
6. Fermentation time: Using all optimized conditions (6:3:1 substrate, 55% moisture, 37 °C, pH 6.5, 10% inoculum), enzyme activity was measured at 12, 24, 36, 48, 60, and 72 h to determine optimal fermentation duration.

##### 2.2.3.2 Box-behnken response surface optimization

Based on single-factor experiment results, three factors showing the most significant effects on enzyme activity (P < 0.01) were selected for optimization: temperature (X_1_), pH (X_2_), and moisture content (X_3_) (Liu et al., 2025; Menhart et al., 2020; Chiang et al., 2025). These factors were studied using a three-factor, three-level Box-Behnken design with the following levels (Benhamiche et al., 2025):

Factor levels:

Temperature (X_1_): 35 °C (−1), 37 °C (0), 39 °C (+1);

pH (X2): 6.0 (−1), 6.5 (0), 7.0 (+1);

Moisture content (X_3_): 50% (−1), 55% (0), 60% (+1)

Fixed parameters (based on single-factor optimization):

Substrate ratio: 6:3:1 (wheat bran: soybean meal: whey powder); Inoculum size: 10% (v/w, OD600 = 1.8 ± 0.1); Fermentation time: 48 h; Total substrate: 10 g.

The substrate ratio (6:3:1) was fixed at the optimal level from single-factor experiment 1. Inoculum size was maintained at 10% as single-factor results showed no significant improvement beyond this level (P > 0.05). Fermentation time was fixed at 48 h based on the kinetic study showing maximum enzyme activity at this time point.

A total of 17 experimental runs were conducted according to the Box-Behnken design matrix, including 5 center points for error estimation and model validation. Enzyme activity (Y, U/g) was the response variable.

##### 2.2.3.3 Data analysis and optimization prediction

Experimental data were analyzed using Design-Expert 8.0 software (Stat-Ease Inc., Minneapolis, United States). A second-order polynomial regression model was established to correlate the relationship between independent variables (*X*_*1*_ *, X*_*2*_ *, X*_*3*_) and the response variable (*Y*):

$$Y=\beta_{0}+\sum \beta_{i}X_{i}+\sum \beta_{ii}X_{i}^{2}+\sum \sum \beta_{ij}X_{i}X_{j}$$

where *Y* is the predicted enzyme activity (U/g); $\beta_{0}$ is the constant coefficient; $\beta_{i}$ , $\beta_{ii}$ , and $\beta_{ij}$ are the linear, quadratic, and interaction coefficients, respectively; and $X_{i}$ and $X_{j}$ are the coded independent variables.

The adequacy and fitness of the model were evaluated through analysis of variance (ANOVA) at a significance level of P < 0.05. Model quality was assessed using the coefficient of determination (*R* ^2^), adjusted *R* ^2^ ( $R_{adj}^{2}$ ), predicted *R* ^2^ ( $R_{pred}^{2}$ ), adequate precision (ratio >4), and lack-of-fit test. Significance of individual coefficients was determined by F-test and P-values. Three-dimensional response surface plots and two-dimensional contour plots were generated to visualize the interactions between variables and identify optimal regions.

Optimal fermentation conditions were predicted by solving the regression equation using the numerical optimization function in Design-Expert software, with the objective of maximizing enzyme activity. The optimization criteria were set to maximize the response variable *Y* within the experimental range of each factor. The software employs a desirability function approach to search for optimal values. Predicted optimal conditions were validated experimentally in triplicate to confirm model accuracy.

#### 2.2.4 Crude enzyme preparation

Solid-state fermentation product was mixed with phosphate buffer (50 mM, pH 6.5) at 1:10 (w/v), extracted by shaking at 30 °C for 2 h (150 rpm). After filtering through double-layer gauze, the filtrate was centrifuged at 10,000×g, 4 °C for 20 min; the supernatant was the crude enzyme solution. Crude enzyme was subjected to 30%–70% saturation ammonium sulfate fractionation, precipitate collected, dialyzed to remove salts, and used for enzymatic property studies (Ruiz-Ramírez and Jiménez-Flores, 2024). For activity and protein quantification, crude enzyme solution (after centrifugation, before ammonium sulfate fractionation) was used to represent the practical enzyme preparation from SSF bioprocesses. This crude extract contains β-galactosidase along with other cellular proteins. The partially purified enzyme (after ammonium sulfate fractionation and dialysis) was used specifically for enzymatic property studies (pH, temperature, kinetics) to characterize the intrinsic properties of the enzyme. This two-tier approach allows assessment of both practical application potential (crude enzyme) and fundamental enzyme characteristics (purified enzyme).

#### 2.2.5 Enzymatic property studies

Optimal pH and pH Stability: Enzyme activity was measured in the pH range of 4.0–9.0 (0.5 intervals) to determine optimal pH. Enzyme solutions were stored in different pH buffers at 4 °C for 24 h, then residual activity was measured to evaluate pH stability.

Optimal Temperature and Thermal Stability: Enzyme activity was measured in the range of 25 °C–60 °C (5 °C intervals) to determine optimal temperature. Enzyme solutions were incubated at different temperatures for 0–6 h, with samples taken at intervals to measure residual activity and evaluate thermal stability (Király et al., 2021).

Effect of Metal Ions: 5 mM of K^+^, Na^+^, Ca^2+^, Mg^2+^, Mn^2+^, Cu^2+^, Zn^2+^, and Fe^3+^ were added separately to the reaction system. Relative activity was calculated with enzyme activity without metal ions as 100%.

Kinetic Parameters: Using different ONPG concentrations (0.5–10 mM) as substrate, initial reaction rates were measured. Michaelis constant (Km) and maximum reaction rate (Vmax) were calculated using Lineweaver-Burk double reciprocal plot (Yan et al., 2021).

#### 2.2.6 Data processing and statistical analysis

All experiments were performed in triplicate, with results expressed as mean ± standard deviation. One-way ANOVA was performed using SPSS 22.0 software, with Duncan’s method for multiple comparisons; P < 0.05 was considered significant. Origin 9.0 software was used for graphing. Response surface analysis was conducted using Design-Expert 8.0 software to establish regression models and optimization (Liu et al., 2025).

#### 2.2.7 Sensory evaluation

Sensory evaluation of low-lactose dairy products was conducted according to detailed protocols provided in Supplementary Material S1, following ISO 13299:2016 and GB/T 16,291.1–2012 standards. The study was conducted in accordance with the Declaration of Helsinki and ethical principles for research involving human subjects. All participants provided written informed consent prior to participation.

## 3 Results and analysis

### 3.1 Isolation and identification of high-producing strains

#### 3.1.1 Strain screening results

A total of 126 lactic acid bacteria strains were isolated from 10 traditional fermented dairy products, including 92 rod-shaped and 34 coccal bacteria. Through X-gal plate primary screening, 45 strains produced distinct blue color on screening medium containing X-gal, indicating β-galactosidase activity. The ratio of blue zone diameter to colony diameter (D/d) for these 45 strains ranged from 1.2 to 3.8. Eighteen strains with D/d > 2.0 were selected for secondary screening.

Secondary screening results showed significant differences in β-galactosidase activity among the 18 strains under liquid fermentation conditions (P < 0.05), with enzyme activities ranging from 12.3 to 44.7 U/g. Strain LP-15, isolated from Tibetan fermented yak milk, showed the best performance with enzyme activity of 44.7 ± 1.2 U/g, significantly higher than other strains (P < 0.05). This was followed by strain LK-8 from Xinjiang kumiss (38.9 ± 0.9 U/g) and strain LM-3 from Inner Mongolia cheese (36.2 ± 1.1 U/g). Compared to the standard strain *L. plantarum* ATCC 8014 (31.5 ± 0.8 U/g), LP-15 showed a 41.9% increase in enzyme activity. Strain LP-15 maintained stable enzyme activity (43.8–45.2 U/g) after 10 successive passages, demonstrating good genetic stability, and was therefore selected for subsequent studies. Protein concentration analysis showed that LP-15 produced 6.5 ± 0.3 mg protein per gram of dry substrate, corresponding to a specific activity of 6.88 ± 0.35 U/mg protein, which was 1.6-fold higher than the median specific activity (4.3 U/mg) of the 18 screened strains.

Notably, strains from fermented dairy products in high-altitude regions (Tibet, Xinjiang) generally exhibited higher β-galactosidase activity, with 11 strains (61.1%) among the high-producing strains originating from these two regions, possibly related to the special selection pressure of high-altitude environments.

#### 3.1.2 Identification of strain LP-15

Morphological Characteristics: Strain LP-15 formed circular, regular-edged, smooth-surfaced, centrally convex milky white colonies on MRS agar plates, with diameters of 1.5–2.0 mm, producing no pigment. Gram staining was positive, with short rod or rod-shaped cells measuring (0.6–0.8) × (2.0–3.5) μm, arranged singly, in pairs, or in short chains, without spores or flagella. Scanning electron microscopy showed smooth cell surfaces with blunt rounded ends, with some cells showing division states.

Detailed methodology for physiological and biochemical characterization is provided in Supplementary Material S2. Physiological and Biochemical Properties: Comprehensive physiological and biochemical characterization of strain LP-15 is presented in Supplementary Table S1. The strain exhibited typical *Lactobacillus* plantarum characteristics: facultatively anaerobic, catalase-negative, and unable to hydrolyze gelatin or reduce nitrate. Carbohydrate fermentation tests showed the strain produced acid from 11 sugars including glucose, galactose, lactose, and maltose, but did not ferment xylose, arabinose, rhamnose, or raffinose. Growth characteristics revealed optimal conditions at pH 6.0–6.5 °C and 37 °C, with pH tolerance range of 4.5–8.0 and temperature range of 15 °C–45 °C. The strain tolerated up to 6.5% NaCl, grew slowly at 10 °C, but did not grow at 45 °C. The arginine hydrolysis test was negative, while citrate utilization was positive. These characteristics were completely consistent with the standard description of *Lactobacillus plantarum* (Refaat et al., 2025).

**FIGURE 2:**
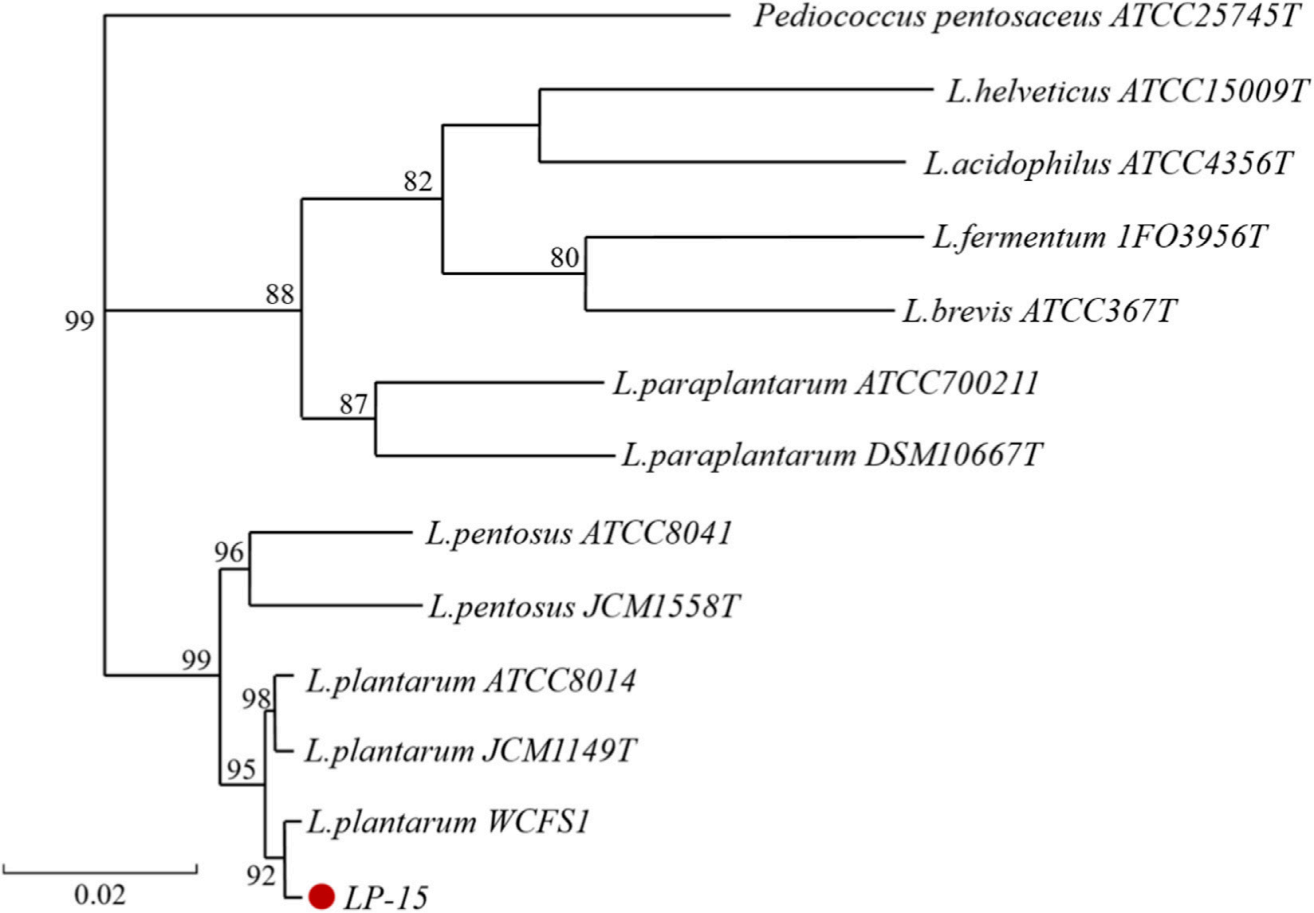
16S rDNA Phylogenetic Tree.

16S rDNA Sequence Analysis: The 16S rDNA sequence of strain LP-15 was 1,487 bp in length. BLAST comparison showed that LP-15 shared over 99.8% similarity with multiple *Lactobacillus plantarum* 16S rDNA sequences, with highest similarity (99.86%) to *L. plantarum* WCFS1 (NR_075041.2) and 99.79% similarity to *L. plantarum* JCM 1149^T^ (type strain). Phylogenetic tree analysis (Figure 2) showed that LP-15 clustered with *L. plantarum* type strain and other known *L. plantarum* strains with a bootstrap value of 99%, clearly separated from closely related species such as *L. pentosus* and *L. paraplantarum*, with genetic distances all greater than 0.02.

Based on the high consistency of morphological, physiological-biochemical properties, and molecular biological identification results, strain LP-15 was identified as *Lactobacillus plantarum*.

#### 3.1.3 Safety evaluation of strain LP-15

Detailed methodology for safety evaluation is provided in Supplementary Material S3. Metabolite Safety: High-performance liquid chromatography detection showed that strain LP-15 did not produce biogenic amines (histamine, tyramine, putrescine, cadaverine, tryptamine, phenylethylamine). After 48 h fermentation in MRS medium, none of the above biogenic amines were detected (detection limit <5 mg/L). Blood agar plate test showed that LP-15 did not produce hemolytic zones, exhibiting γ-hemolysis (no hemolysis), while the positive control *Staphylococcus aureus* produced distinct β-hemolytic zones (Binda et al., 2020; Refaat et al., 2025).

Antibiotic Sensitivity: Minimum inhibitory concentrations (MICs) of LP-15 for 12 antibiotics were determined using the broth microdilution method. Results showed the strain was sensitive to ampicillin (2 μg/mL), penicillin G (1 μg/mL), erythromycin (1 μg/mL), tetracycline (4 μg/mL), chloramphenicol (4 μg/mL), clindamycin (1 μg/mL), gentamicin (16 μg/mL), and kanamycin (32 μg/mL), with MIC values all below EFSA-recommended breakpoints. It showed intrinsic resistance to vancomycin (>256 μg/mL), a typical characteristic of Gram-positive *Lactobacillus*. PCR detection found no transferable antibiotic resistance genes such as ermB, ermC, tetM, or tetS (Refaat et al., 2025).

Cell Adhesion: LP-15 adhesion capacity was evaluated using Caco-2 human colon cancer cell line. Results showed LP-15 adhesion rate to Caco-2 cells was (8.3 ± 0.6)%, comparable to commercial probiotic *L. plantarum* 299v (7.8 ± 0.5)% and significantly higher than *L. plantarum* ATCC 8014 (4.2 ± 0.3)%. Moderate adhesion favors intestinal colonization without causing adverse reactions from excessive adhesion.

Tolerance Evaluation: After 2 h treatment in artificial gastric juice (pH 2.5, containing pepsin), LP-15 survival rate was 68.5% ± 3.2%; after 4 h treatment in artificial intestinal juice (pH 8.0, containing trypsin), survival rate was 75.3% ± 2.8%. It showed good tolerance to 0.3% bile salts, with viable counts decreasing only 0.35 log units after 4 h growth in MRS medium containing 0.3% ox bile.

These *in vitro* safety evaluation results demonstrate that strain LP-15 does not produce harmful metabolites, has appropriate antibiotic sensitivity, possesses good probiotic properties, and meets safety requirements for food industry strains.

### 3.2 Optimization of solid-state fermentation conditions

#### 3.2.1 Single-factor experiment results

**FIGURE 3:**
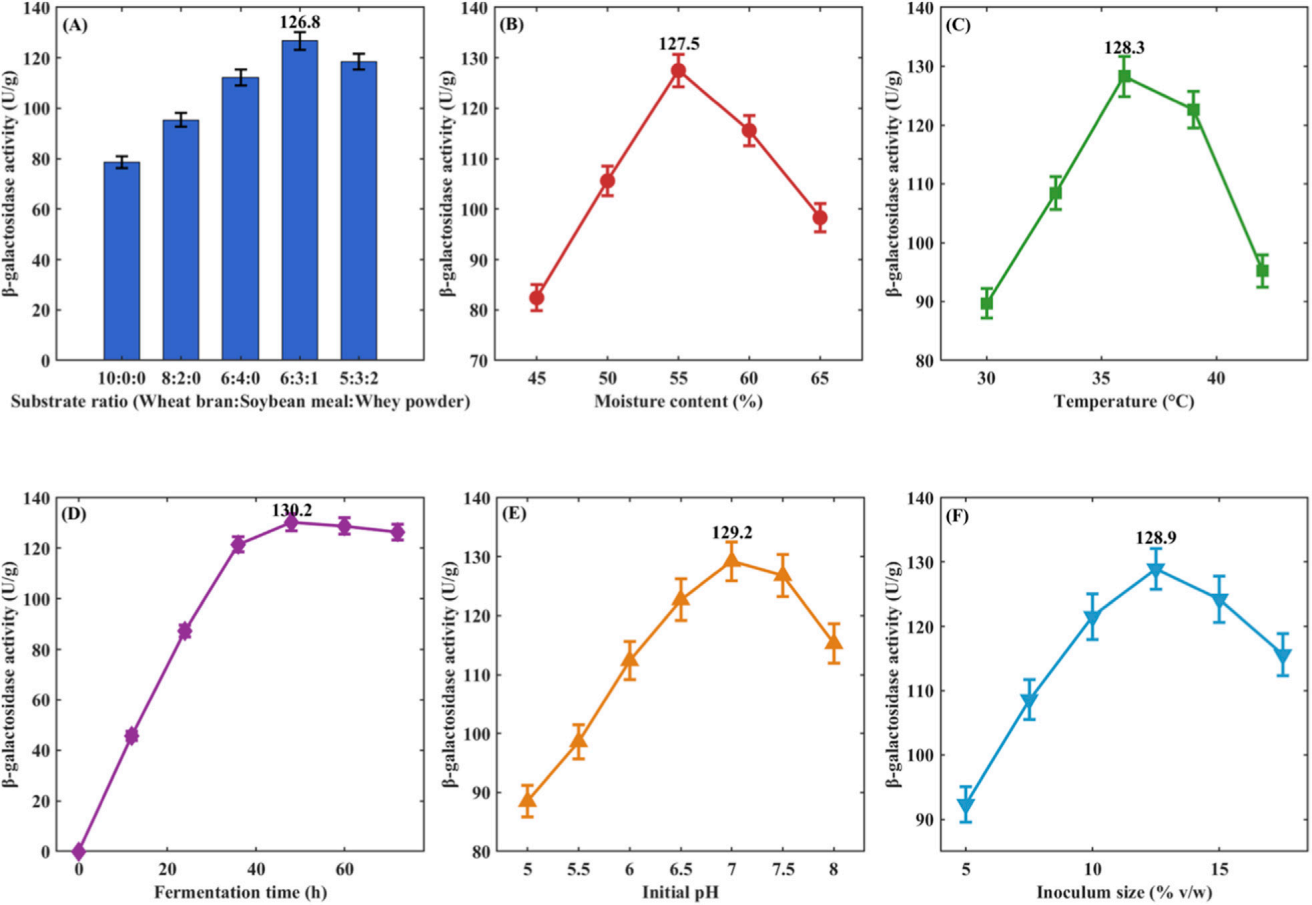
Single-factor optimization of solid-state fermentation conditions. **(A)** Effect of substrate composition; **(B)** Effect of moisture content; **(C)** Effect of temperature; **(D)** Effect of fermentation time. Error bars represent standard deviation (n = 3); **(E)** Effect of pH; **(F)** Effect of inoculum size.

Effect of Substrate Ratio: Different substrate Ratios significantly affected enzyme activity (P < 0.05) (Figure 3A). Pure wheat bran as substrate yielded enzyme activity of 78.6 ± 2.3 U/g, wheat bran: soybean meal = 6:4 increased it to 112.3 ± 3.1 U/g, and wheat bran: soybean meal: whey powder = 6:3:1 achieved the highest value of 126.8 ± 3.5 U/g. Further increases in whey powder ratio decreased enzyme activity. The ratio 6:3:1 was selected as the optimal substrate composition.

The effect of moisture content on enzyme activity is shown in Figure 3B. As moisture content increased from 45% to 55%, enzyme activity increased from 82.4 ± 2.6 U/g to 126.8 ± 3.2 U/g; further increases to 60% and 65% reduced enzyme activity to 115.6 ± 3.0 U/g and 98.3 ± 2.8 U/g, respectively. Excessive moisture impeded oxygen transfer. 55% was determined as the optimal moisture content.

Temperature optimization results are shown in Figure 3C. Enzyme activity was 89.7 ± 2.5 U/g at 30 °C, reached maximum at 37 °C (128.3 ± 3.4 U/g), and decreased to 122.6 ± 3.1 U/g and 95.2 ± 2.7 U/g at 40 °C and 43 °C, respectively. 37 °C was selected as the optimal temperature.

pH significantly affected enzyme activity (Figure 3C). Maximum enzyme activity (129.2 ± 3.3 U/g) occurred at pH 6.5, decreasing to 76.8 ± 2.2 U/g and 84.6 ± 2.4 U/g at pH 5.0 and 8.0, respectively. pH 6.5 was determined as optimal.

Effect of Inoculum Size: Maximum enzyme activity (128.9 ± 3.2 U/g) was achieved with 10% inoculum, compared to only 98.6 ± 2.7 U/g at 5% and 119.7 ± 3.0 U/g at 15%. 10% was selected as optimal inoculum size.

The fermentation time course curve (Figure 3D) showed enzyme activity peaked at 48 h (130.2 ± 3.4 U/g), with slight decreases at 60 h and 72 h. 48 h was determined as optimal fermentation time.

pH significantly affected enzyme activity (Figure 3E). Maximum enzyme activity (129.2 ± 3.3 U/g) occurred at pH 6.5, decreasing to 76.8 ± 2.2 U/g and 84.6 ± 2.4 U/g at pH 5.0 and 8.0, respectively. The optimal pH range for L. plantarum LP-15 growth and enzyme production was 6.0–7.0, consistent with typical lactic acid bacteria characteristics. pH 6.5 was determined as optimal for subsequent response surface optimization.

Effect of Inoculum Size: Maximum enzyme activity (128.9 ± 3.2 U/g) was achieved with 10% inoculum (Figure 3F), compared to only 98.6 ± 2.7 U/g at 5% and 119.7 ± 3.0 U/g at 15%. Lower inoculum size resulted in insufficient bacterial density for optimal enzyme production, while excessive inoculum led to rapid nutrient depletion and premature entry into stationary phase. 10% was selected as optimal inoculum size for response surface optimization.

#### 3.2.2 Response surface optimization results

Based on single-factor results, temperature (X_1_: 35 °C–39 °C), pH (X_2_: 6.0–7.0), and moisture content (X_3_: 50%–60%) were selected for Box-Behnken design (Table 1). Enzyme activities in 17 experimental runs ranged from 142.3 to 186.3 U/g, with good reproducibility at center points (186.3 ± 2.1 U/g).

**TABLE 1:** Box-behnken experimental design and response surface analysis results.

Run	Coded levels			Actual levels			Enzyme activity (U/g)		Error (%)
	X_1_	X_2_	X_3_	Temp (°C)	pH	Moisture (%)	Observed	Predicted	
1	−1	−1	0	35	6.0	55	142.3 ± 1.8	143.1	0.56
2	+1	−1	0	39	6.0	55	156.7 ± 2.1	155.9	0.51
3	−1	+1	0	35	7.0	55	148.5 ± 1.9	149.3	0.54
4	+1	+1	0	39	7.0	55	164.2 ± 2.2	163.4	0.49
5	−1	0	−1	35	6.5	50	145.6 ± 1.9	144.8	0.55
6	+1	0	−1	39	6.5	50	158.9 ± 2.1	159.7	0.50
7	−1	0	+1	35	6.5	60	151.3 ± 2.0	150.5	0.53
8	+1	0	+1	39	6.5	60	165.8 ± 2.2	166.6	0.48
9	0	−1	−1	37	6.0	50	152.4 ± 2.0	151.6	0.52
10	0	+1	−1	37	7.0	50	159.7 ± 2.1	160.5	0.50
11	0	−1	+1	37	6.0	60	168.9 ± 2.3	168.1	0.47
12	0	+1	+1	37	7.0	60	173.2 ± 2.4	174.0	0.46
13	0	0	0	37	6.5	55	186.3 ± 2.5	185.7	0.32
14	0	0	0	37	6.5	55	185.8 ± 2.4	185.7	0.05
15	0	0	0	37	6.5	55	186.7 ± 2.5	185.7	0.54
16	0	0	0	37	6.5	55	185.2 ± 2.4	185.7	0.27
17	0	0	0	37	6.5	55	187.1 ± 2.5	185.7	0.75

Regression Model:

$$\begin{array}{c} Y=186.3+8.2X_{1}+6.7X_{2}+5.3X_{3}-12.4X_{1}^{2}-9.8X_{2}^{2}-7.6X_{3}^{2}\\ +4.2X_{1}X_{2}+3.1X_{1}X_{3}+2.8X_{2}X_{3} \end{array}$$

**FIGURE 4:**
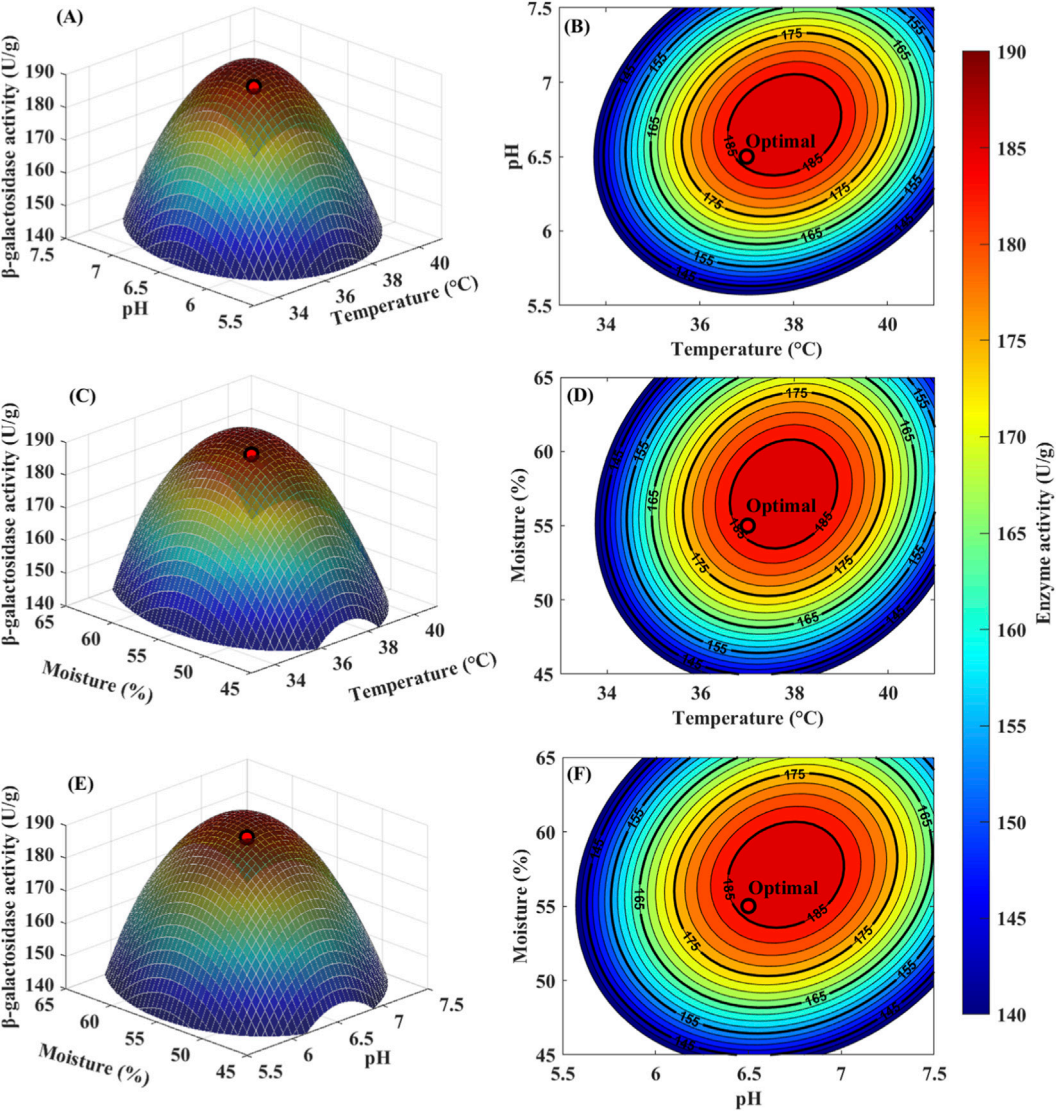
Response surface and contour plots showing factor interactions on B-galactosidase activity **(A,B)** Temperature x pH (moisture = 55%); **(C,D)** Temperature x Moisture (pH = 6.5); **(E,F)** pH x Moisture (temp = 37 °C).

The model showed excellent statistical significance (F = 61.85, P < 0.0001) with high predictive capability (*R* ^2^ = 0.9876, Adjusted *R* ^2^ = 0.9716, Predicted *R* ^2^ = 0.9432). Complete analysis of variance (ANOVA) with detailed regression coefficients, standard errors, t-values, and comprehensive model diagnostics are presented in Supplementary Table S2. All linear terms (X_1_, X_2_, X_3_), quadratic terms (X_1_^2^, X_2_^2^, X_3_^2^), and interaction terms (X_1_X_2_, X_1_X_3_, X_2_X_3_) were statistically significant (P < 0.01), demonstrating their critical roles in enzyme production optimization. The X_1_X_2_ interaction (temperature × pH) was highly significant (P = 0.0003), as were X_1_X_3_ (P = 0.0016) and X_2_X_3_ (P = 0.0028) interactions. The lack-of-fit test was not significant (F = 1.05, P = 0.4523), confirming the model adequately describes the experimental data. The root mean square error (RMSE) of 2.48 U/g and adequate precision of 24.58 (>>4) demonstrated excellent model precision and discrimination capability. Response surface plots (Figure 4) illustrated all three pairwise factor interactions. The temperature-pH interaction (Figures 4A,B) showed distinct elliptical contours. The temperature-moisture interaction (Figures 4C,D) revealed optimal activity within 36 °C–38 °C and 52%–58% moisture content. The pH-moisture interaction (Figures 4E,F) exhibited broader tolerance when pH was near optimum (6.0–7.0). All interactions were statistically significant (p < 0.01, Supplementary Table S1), validating the Box-Behnken optimization approach.

Optimal Condition Validation: Predicted optimal conditions were temperature 37.2 °C, pH 6.48, moisture content 55.3%, with predicted enzyme activity 187.5 ± 1.5 U/g (standard error of prediction). Actual operating conditions were adjusted to 37 °C, pH 6.5, moisture 55%, with validation experiments yielding enzyme activity of 186.3 ± 2.1 U/g and specific activity of 12.26 ± 0.62 U/mg protein, an error of 0.64% from predicted value. Optimized enzyme activity increased 4.17-fold and specific activity increased 1.78-fold compared to initial values (44.7 U/g and 6.88 U/mg protein, respectively).

Comprehensive comparison of enzyme production parameters before and after optimization is presented in Table 2. The data clearly demonstrate that the 4.17-fold improvement in volumetric activity resulted from both enhanced protein synthesis (2.34-fold increase in protein concentration) and improved enzyme quality (1.78-fold increase in specific activity). This dual improvement indicates that the optimized conditions not only promoted cell growth and total protein production but also enhanced the intrinsic catalytic properties of the enzyme.

**TABLE 2:** Comparison of enzyme production before and after optimization.

Parameter	Before optimization	After optimization	Improvement factor
Fermentation conditions			
Substrate ratio (W:S:W)*	10:0:0	6:3:1	Optimized
Temperature (°C)	37	37	-
pH	6.5	6.5	-
Moisture content (%)	50	55	+10%
Enzyme production metrics			
Volumetric activity (U/g)	44.7 ± 1.2	186.3 ± 2.1	4.17-fold
Protein concentration (mg/g)	6.5 ± 0.3	15.2 ± 0.8	2.34-fold
Specific activity (U/mg)	6.88 ± 0.35	12.26 ± 0.62	1.78-fold
Process efficiency			
Protein yield (mg/g substrate)	6.5	15.2	2.34-fold
Enzyme productivity (U/g/h)	0.93	3.88	4.17-fold

*W:S:W = wheat bran:soybean meal:whey powder; Values expressed as mean ± standard deviation (n = 5); Volumetric activity refers to enzyme units per gram of initial dry substrate; specific activity refers to enzyme units per milligram of total crude protein. The greater improvement in volumetric activity (4.17-fold) compared to specific activity (1.78-fold) indicates that optimization primarily enhanced total protein synthesis and enzyme production capacity, while also improving enzyme quality.

### 3.3 Enzymatic property characterization

The key property parameters of β-galactosidase produced by *L. plantarum* LP-15 are summarized in Table 3. The enzyme showed maximum activity at pH 6.5 °C and 42 °C, stability in pH range 5.0–8.0, maintained activity for over 4 h at 40 °C, activation by Ca^2+^ and Mg^2+^, Km value of 2.8 mM, and catalytic efficiency of 1.2 × 10^5^ M^-1^s^-1^ (Ruiz-Ramírez and Jiménez-Flores, 2024; Király et al., 2021; Yan et al., 2021; Duan et al., 2022). These properties indicate the enzyme is highly suitable for dairy processing applications (Costa et al., 2024).

**TABLE 3:** Key Property Parameters of β-Galactosidase.

Parameter category	Parameter	Value/Range	Notes
Optimal conditions			
	pH	6.5	100% relative activity
	Temperature	42 °C	100% relative activity
Stability range			
	pH stability	5.0–8.0	>80% activity, stored at 4 °C for 24 h
	Thermal stability	40 °C, 4 h	>85% activity
		45 °C, 4 h	58.3% activity
		50 °C, 2 h	31.5% activity
Kinetic parameters			
	Km (ONPG)	2.8 ± 0.1 mM	Substrate affinity
	Km (lactose)	18.5 ± 0.5 mM	Natural substrate
	Vmax	215.6 ± 5.3 μmol/min/mg	Maximum reaction rate
	Kcat	148.2 s^-1^	Turnover number
	kcat/Km	5.3 × 10^4^ M^-1^s^-1^	Catalytic efficiency
Metal ion effects			5 mM concentration
	Activation		
	Ca^2+^	125.3% ± 3.2%	
	Mg^2+^	118.6% ± 2.8%	
	Mn^2+^	112.4% ± 2.5%	
	No significant effect		
	K^+^	103.2% ± 1.8%	
	Na^+^	98.7% ± 2.1%	
	Inhibition		
	Cu^2+^	45.2% ± 1.5%	
	Fe^3+^	58.4% ± 2.0%	
	Zn^2+^	62.7% ± 1.9%	
Product inhibition			
	Ki (galactose)	85 mM	Competitive inhibition
	Ki (glucose)	320 mM	Competitive inhibition

Relative activity calculated with activity under optimal conditions as 100%; Values expressed as mean ± standard deviation (n = 3); ONPG: o-nitrophenyl-β-D-galactopyranoside.

#### 3.3.1 Effect of pH on enzyme activity

β-Galactosidase activity varied significantly under different pH conditions. Within pH range 4.0–9.0, enzyme activity showed an initial increase followed by decrease. Relative activity was only 32.5% at pH 4.0, gradually increasing with pH to reach maximum (defined as 100%) at pH 6.5, maintaining 92.3% activity at pH 7.0, but decreasing to 68.7% and 41.2% at pH 8.0 and 9.0, respectively.

pH stability studies showed good enzyme stability in pH range 5.0–8.0. After storage at 4 °C for 24 h, enzyme activity remained above 85% in pH range 6.0–7.0, with 80.3% and 81.6% activity retained at pH 5.0 and 8.0, respectively. Stability was poor at pH 4.0 and 9.0, with residual activities of 45.6% and 38.9%, respectively. The enzyme’s pH characteristics closely match milk (pH 6.6–6.8), favoring practical application.

#### 3.3.2 Effect of temperature on enzyme activity

Temperature significantly affected enzyme activity. Relative activity was 48.6% at 25 °C, increasing with temperature to reach maximum (100%) at 42 °C, maintaining 94.8% at 45 °C, decreasing to 76.3% and 52.4% at 50 °C and 55 °C respectively, and only 28.7% at 60 °C.

Thermal stability tests showed the enzyme retained 85.2% activity after 4 h at 40 °C, and 78.6% after 6 h. Stability decreased at 45 °C, with 58.3% residual activity after 4 h. Activity decreased to 31.5% after 2 h treatment at 50 °C. The enzyme would be inactivated at milk pasteurization temperature (63 °C, 30 min) but remained stable at moderate processing temperatures (40 °C–45 °C), suitable for enzymatic lactose hydrolysis in dairy applications.

#### 3.3.3 Effect of metal ions

Different metal ions (5 mM) had varying effects on enzyme activity. Ca^2+^ and Mg^2+^ showed activation effects, with relative activities of 125.3% and 118.6% respectively; Mn^2+^ had slight activation effect (112.4%); K^+^ and Na^+^ had minimal effects, with relative activities of 103.2% and 98.7% respectively.

Heavy metal ions showed varying degrees of inhibition: Cu^2+^ showed strongest inhibition (relative activity 45.2%), followed by Fe^3+^ (58.4%) and Zn^2+^ (62.7%). EDTA (5 mM) treatment reduced enzyme activity to 73.5%, suggesting the enzyme may be a metalloenzyme. Ca^2+^ activation is advantageous for practical applications, as milk is rich in calcium ions (approximately 1,200 mg/L).

#### 3.3.4 Kinetic parameters

Using ONPG as substrate, initial reaction rates were measured at different substrate concentrations (0.5–10 mM) under optimal conditions. Lineweaver-Burk double reciprocal plot showed good linearity (*R* ^2^ = 0.9956).

Kinetic parameters were as follows: Km = 2.8 ± 0.1 mM, indicating high enzyme-substrate affinity; Vmax = 215.6 ± 5.3 μmol/min/mg protein; catalytic constant kcat = 148.2 s^-1^; catalytic efficiency kcat/Km = 5.3 × 10^4^ M^-1^s^-1^. Compared to other reported lactic acid bacterial β-galactosidases, the enzyme from LP-15 had lower Km value (most are 3–7 mM) and higher catalytic efficiency, indicating good catalytic performance.

Substrate specificity studies showed the enzyme’s Km for lactose was 18.5 mM, higher than for ONPG, a common characteristic of β-galactosidases. Product inhibition experiments showed Ki values of 85 mM for galactose and 320 mM for glucose, indicating galactose as the primary competitive inhibitor.

## 4 Application in low-lactose dairy products

### 4.1 Optimization of lactose hydrolysis process

#### 4.1.1 Optimization of hydrolysis conditions

**FIGURE 5:**
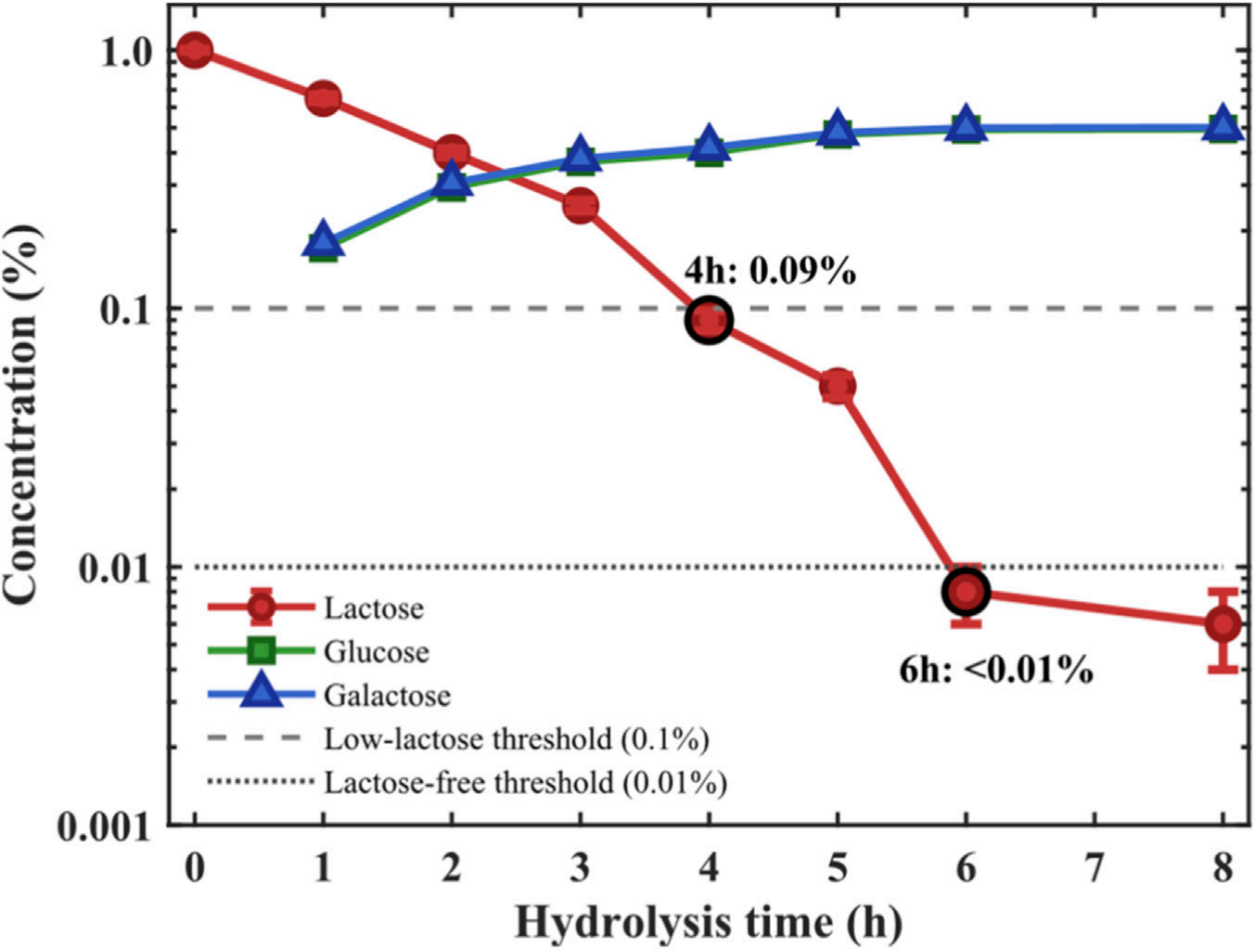
Kinetics of lactose hydrolysis in whole milk.

The effects of enzyme dosage, temperature, and time on lactose hydrolysis were systematically optimized (Schulz and Rizvi, 2023; Harwood and Drake, 2020). Enzyme dosage experiments showed 2.0 U/mL as optimal, achieving 81.9% hydrolysis in 4 h and 95.2% in 6 h; increasing enzyme dosage to 3.0 U/mL only improved hydrolysis to 96.8%. Lactose hydrolysis kinetics (Figure 5) showed that at 2.0 U/mL, lactose could be reduced to 0.09% (meeting the low-lactose standard of ≤0.1% according to EU Regulation No 1169/2011) in 4 h and to <0.01% (meeting the lactose-free standard of ≤0.01% according to EU Regulation No 1169/2011 and Codex Alimentarius) in 6 h (Zheng et al., 2023; Portnoy and Barbano, 2021).

Temperature optimization results showed highest hydrolysis efficiency at 40 °C (82.6% in 4 h), higher than at 35 °C (68.4%) and 45 °C (78.9%), consistent with enzyme thermal stability results. The original pH of whole milk (6.65 ± 0.05) was close to the enzyme’s optimal pH, with no significant difference between direct hydrolysis and pH-adjusted hydrolysis (82.6% vs. 83.2%, P > 0.05), thus pH adjustment was unnecessary.

#### 4.1.2 Application in different dairy products

The optimized process was applied to different dairy products with results shown in Table 4. Whole milk under standard conditions (2.0 U/mL, 40 °C) achieved hydrolysis rates of 81.9% ± 1.2% at 4 h (residual lactose 0.87%, near low-lactose standard) and 99.2% ± 0.5% at 6 h (residual lactose 0.04%, meeting lactose-free standard of <0.01% according to EU Regulation No 1169/2011).

**TABLE 4:** Time course of enzymatic lactose hydrolysis in whole milk and skim milk.

Treatment time (h)	Initial lactose (g/100 mL)	Residual lactose		Hydrolysis products (g/100 mL)		Hydrolysis rate (%)	Product type
		(g/100 mL)	(%)	Glucose	Galactose		
Whole milk							
0	4.80 ± 0.05	4.80 ± 0.05	100.0	ND	ND	0	Regular milk
1	4.80 ± 0.05	3.12 ± 0.08	65.0	0.82 ± 0.03	0.86 ± 0.03	35.0 ± 1.5	Partially hydrolyzed
2	4.80 ± 0.05	1.92 ± 0.06	40.0	1.41 ± 0.04	1.47 ± 0.04	60.0 ± 1.8	Partially hydrolyzed
3	4.80 ± 0.05	1.20 ± 0.04	25.0	1.77 ± 0.05	1.83 ± 0.05	75.0 ± 2.0	Partially hydrolyzed
4	**4.80 ± 0.05**	**0.87 ± 0.03**	**18.1**	**1.92 ± 0.05**	**2.01 ± 0.05**	**81.9 ± 1.2**	**Near low-lactose**
5	4.80 ± 0.05	0.24 ± 0.01	5.0	2.26 ± 0.06	2.30 ± 0.06	95.0 ± 0.8	Low-lactose
6	**4.80 ± 0.05**	**0.04 ± 0.01**	**0.8**	**2.36 ± 0.07**	**2.40 ± 0.07**	**99.2 ± 0.5**	**Lactose-free (<0.01%)**
8	4.80 ± 0.05	0.03 ± 0.01	0.6	2.37 ± 0.07	2.40 ± 0.07	99.4 ± 0.4	Lactose-free
Skim milk							
0	4.76 ± 0.04	4.76 ± 0.04	100.0	ND	ND	0	Regular milk
1	4.76 ± 0.04	2.95 ± 0.07	62.0	0.89 ± 0.03	0.92 ± 0.03	38.0 ± 1.6	Partially hydrolyzed
2	4.76 ± 0.04	1.76 ± 0.05	37.0	1.47 ± 0.04	1.53 ± 0.04	63.0 ± 1.7	Partially hydrolyzed
3	4.76 ± 0.04	1.05 ± 0.04	22.1	1.83 ± 0.05	1.88 ± 0.05	77.9 ± 1.9	Partially hydrolyzed
**4**	**4.76 ± 0.04**	**0.65 ± 0.03**	**13.7**	**2.03 ± 0.06**	**2.08 ± 0.06**	**86.3 ± 1.3**	**Low-lactose**
5	4.76 ± 0.04	0.18 ± 0.01	3.8	2.27 ± 0.06	2.31 ± 0.06	96.2 ± 0.7	Low-lactose
**6**	**4.76 ± 0.04**	**0.02 ± 0.01**	**0.4**	**2.35 ± 0.07**	**2.39 ± 0.07**	**99.6 ± 0.4**	**Lactose-free (<0.01%)**
8	4.76 ± 0.04	0.01 ± 0.00	0.2	2.36 ± 0.07	2.39 ± 0.07	99.8 ± 0.3	Lactose-free

Enzyme dosage: 2.0 U/mL; Temperature: 40 °C; pH: 6.65 (milk original pH); ND: Not detected; Low-lactose standard: residual lactose ≤ 0.1 g/100 mL (≤0.1%) according to EU Regulation No 1169/2011; Lactose-free standard: residual lactose ≤ 0.01 g/100 mL (≤0.01%) according to EU Regulation No 1169/2011 and Codex Alimentarius; Values expressed as mean ± standard deviation (n = 3); Bold values indicate key time points where products reached commercial standards: 4 h for low-lactose standard (residual lactose ≤0.1%) and 6 h for lactose-free standard (residual lactose ≤0.01%). Skim milk showed higher hydrolysis eficiency than whole milk due tolower fat content (0.19% vs. 3.5%) facilitating enzyme-substrate contact and reducing mass transfer resistance.

Skim milk showed higher hydrolysis efficiency, achieving 86.3% ± 1.3% and 99.6% ± 0.4% at 4 h and 6 h respectively, due to lower fat content (0.1%) facilitating enzyme-substrate contact. At 4 h, skim milk reached the low-lactose standard (0.65% residual lactose), while whole milk approached this threshold (0.87%). Both products achieved lactose-free standard by 6 h with excellent color and flavor characteristics maintained.

In yogurt production, pre-fermentation hydrolysis (40 °C, 4 h) was superior to post-fermentation hydrolysis, with final product lactose <0.05% and uniform texture; post-fermentation hydrolysis required pH adjustment and increased enzyme dosage, and resulted in thinner texture (Dantas et al., 2021). Concentrated milk (2×) required 4.0 U/mL enzyme dosage and 8 h hydrolysis time to achieve low-lactose standard.

### 4.2 Product quality evaluation

#### 4.2.1 Physicochemical analysis

Hydrolysis treatment did not affect milk nutritional components, with protein (3.2 ± 0.1 g/100 mL), fat (3.5 ± 0.1 g/100 mL), and calcium (120 ± 3 mg/100 mL) content remaining unchanged (Portnoy and Barbano, 2021). HPLC analysis showed lactose decreased from 4.80 to 0.87 g/100 mL after 4 h hydrolysis, producing 1.92 g/100 mL glucose and 2.01 g/100 mL galactose; after 6 h hydrolysis, lactose decreased to 0.04 g/100 mL pH decreased slightly (6.65→6.55), viscosity decreased slightly (2.15→2.05 mPa·s), with no significant color change (ΔE < 1.0).

#### 4.2.2 Sensory evaluation

**FIGURE 6:**
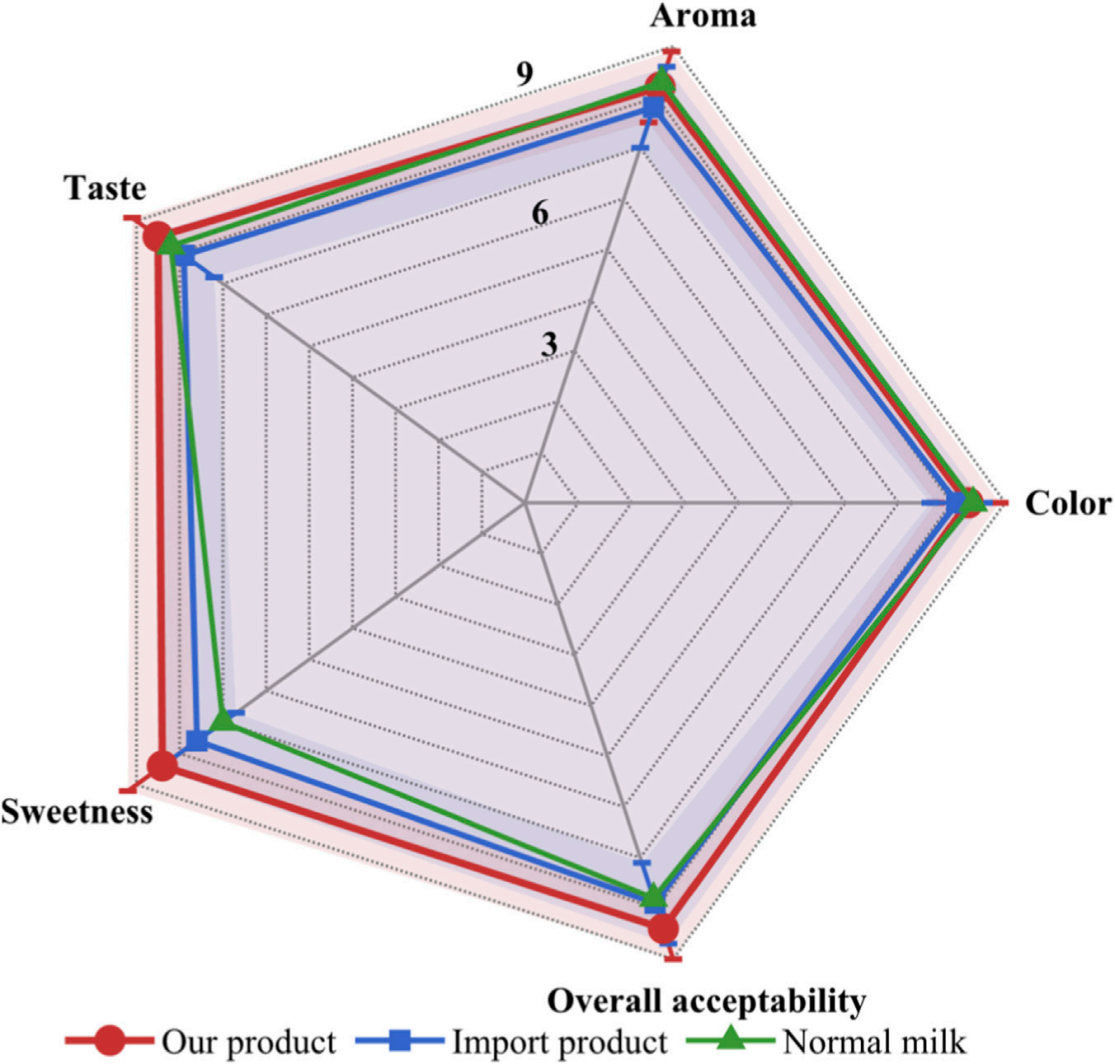
Spider plot of sensory evaluation for low-lactose milk products (n = 100).

Sensory evaluation was conducted with initially recruited 115 consumers. After excluding 10 outliers and 5 inconsistent panelists, 100 consumers (including 60 lactose-intolerant individuals) were included in the final analysis (Figure 6). The results showed low-lactose milk received high scores for color (8.3 ± 0.9), aroma (8.2 ± 1.0), taste (8.5 ± 0.8), sweetness (8.4 ± 0.9), and overall acceptability (8.4), superior to imported control products (commercial brands from Europe, lactose content <0.1%, overall acceptability 7.9 ± 1.2, P < 0.05). Score distributions for all attributes showed unimodal patterns with majority of ratings concentrated in the 7-9 range, indicating consistent consumer preference without polarization. 96.7% of lactose-intolerant individuals experienced no discomfort after consumption, with 93.3% expressing willingness to purchase.

#### 4.2.3 Microbiological indicators

Product microbiological indicators fully complied with GB 19301-2010 standards: total plate count <10 CFU/mL (standard ≤30,000); coliform bacteria and pathogenic bacteria not detected. Membrane-filtered sterilized enzyme preparations and closed hydrolysis system ensured product safety.

#### 4.2.4 Shelf-life study

Pasteurized low-lactose milk maintained stable quality during 14-day storage at 4 °C, with no lactose reversion (maintained at 0.08%–0.09%) and sensory scores above 8.0. UHT lactose-free milk stored at 25 °C for 6 months maintained lactose at <0.01%, with slight Maillard reaction appearing at month 4 but sensory quality still acceptable (overall acceptability: 7.6 ± 0.4 on 9-point scale). Tetra Pak packaging showed best stability and is recommended.

## 5 Techno-economic analysis

### 5.1 Production cost analysis

#### 5.1.1 Raw material cost comparison

Solid-state fermentation using agricultural byproducts as main raw materials showed significant cost advantages (Artola et al., 2024; Balina et al., 2023). Raw material formula was wheat bran: soybean meal: whey powder = 6:3:1, with wheat bran at 0.8 RMB/kg, soybean meal at 3.2 RMB/kg, whey powder at 18 RMB/kg, yielding comprehensive raw material cost of 8.5 RMB/kg dry substrate. Liquid fermentation using MRS medium containing peptone (280 RMB/kg), beef extract (120 RMB/kg), yeast extract (65 RMB/kg), etc., had preparation cost of 25.3 RMB/kg. Solid-state fermentation reduced raw material costs by 66.4%.

Enzyme yield per kg dry substrate: solid-state fermentation 186.3 KU, liquid fermentation 90.5 KU (calculated at 30% yield, based on typical downstream processing efficiency for microbial enzymes including centrifugation, filtration, and concentration steps (Artola et al., 2024; Balina et al., 2023)). Unit enzyme activity raw material cost: SSF 0.046 RMB/KU, SmF 0.280 RMB/KU, a reduction of 83.6%.

#### 5.1.2 Production process costs

Energy Consumption Comparison: Solid-state fermentation employed static cultivation requiring only temperature control, with energy consumption of 0.8 kWh/kg product; liquid fermentation required continuous stirring and aeration, with energy consumption of 2.1 kWh/kg. At industrial electricity rate of 0.7 RMB/kWh (standard industrial tariff in Eastern China (Bradford, 1976)), SSF energy cost was 0.032 RMB/KU, SmF was 0.053 RMB/KU, a reduction of 39.6%.

Water Resource Consumption: Solid-state fermentation only used water for substrate moistening (55% moisture content) and product extraction, with water usage of 1.5 L/kg substrate; liquid fermentation medium contained 95% water, plus washing water, with total water usage of 25 L/kg medium. Water cost (including wastewater treatment): SSF 0.018 RMB/KU, SmF 0.028 RMB/KU, a reduction of 35.7%. Costs calculated based on water supply rate of 4 RMB/ton and wastewater treatment rate of 8 RMB/ton (Eastern China industrial rates (Bradford, 1976)).

Labor Cost: Both processes had comparable operational complexity, with labor cost of 0.025 RMB/KU. Though solid-state fermentation required more effort in loading and unloading, the fermentation process required no monitoring; liquid fermentation required continuous monitoring of pH, dissolved oxygen, and other parameters.

Waste Treatment: Solid-state fermentation residue could be used as feed or organic fertilizer (Pandey et al., 1999), with treatment cost of 0.012 RMB/KU; liquid fermentation produced large amounts of high-COD wastewater (COD >10,000 mg/L), requiring biological treatment (based on aerobic treatment efficiency and sludge disposal costs (Singh et al., 2016)), with treatment cost of 0.024 RMB/KU, a reduction of 50%.

#### 5.1.3 Comprehensive cost analysis

Production cost comparison for annual production of 100 tons of β-galactosidase (enzyme activity 150 KU/g) is shown in Table 5. Solid-state fermentation total cost was 0.180 RMB/KU, liquid fermentation 0.280 RMB/KU, with comprehensive cost reduction of 35.7%. Raw material cost showed the largest reduction (66.4%), followed by waste treatment (50%) and energy consumption (39.6%).

**TABLE 5:** Techno-economic comparison of solid-state and liquid fermentation.

Item	SSF	SmF	Difference
Production cost (RMB/KU)			
Raw material cost	0.085 (47.2%)	0.280 (100.0%)	−66.4%
Energy cost	0.032 (17.8%)	0.053 (18.9%)	−39.6%
Water resource cost	0.018 (10.0%)	0.028 (10.0%)	−35.7%
Labor cost	0.025 (13.9%)	0.025 (8.9%)	0.0%
Equipment depreciation	0.008 (4.4%)	0.007 (2.5%)	+14.3%
Waste treatment	0.012 (6.7%)	0.024 (8.6%)	−50.0%
**Total production cost**	0.180 (100%)	0.280 (100%)	−35.7%
Economic indicators (100 tons/year)			
Fixed asset investment (10^4^ RMB)	2,500	3,800	−34.2%
Annual production cost (10^4^ RMB)	2,700	4,200	−35.7%
Annual sales revenue (10^4^ RMB)	7,500	7,500	0.0%
Annual net profit (10^4^ RMB)	4,000	2,500	+60.0%
Gross profit margin (%)	64.0	44.0	+20.0%
Payback period (years)	2.5	4.2	−40.5%
Internal rate of return IRR (%)	28.0	16.5	+69.7%
Environmental indicators			
Water usage (L/kg product)	1.5	25.0	−94.0%
Wastewater generation (L/kg)	0.8	20.0	−96.0%
COD emission (kg/year)	120	720	−83.3%
CO_2_ emission (tons/year)	350	930	−62.4%
Solid waste utilization rate (%)	95	35	+171.4%

KU: Kilo-unit of enzyme activity; Percentages in parentheses indicate proportion of total cost; Selling price: 50 RMB/KU; Electricity price: 0.7 RMB/kWh; Water price: 12 RMB/ton; Difference column: negative values indicate SSF reduction compared to SmF, positive values indicate increase.

At annual production scale of 100 tons, solid-state fermentation annual production cost was 27 million RMB, liquid fermentation 42 million RMB, with annual cost savings of 15 million RMB. With product pricing at 50 RMB/KU, solid-state fermentation gross profit margin was 64%, liquid fermentation 44%.

Economic Analysis: Data Sources and Sensitivity.

All cost data were collected in June 2024 from typical market prices in eastern China: wheat bran ¥1.2/kg, soybean meal ¥3.5/kg, whey powder ¥8.5/kg, electricity ¥0.65/kWh. Equipment depreciation used 10-year straight-line method with 85% utilization rate. Sensitivity analysis showed that SSF maintains cost advantage over SmF (¥60/kg) even when raw material costs increase by 35%, enzyme activity decreases by 25%, or operating costs increase by 30%, demonstrating robust economic competitiveness across a wide range of market conditions.

### 5.2 Industrial feasibility assessment

#### 5.2.1 Technical feasibility

##### 5.2.1.1 Equipment requirements

Solid-state fermentation employed tray-type or drum-type reactors, with single 10 m^3^ reactor investment of 800,000 RMB, capable of daily production of 500 kg enzyme preparation. Compared to liquid fermentation stainless steel tanks (1.5 million RMB/10 m^3^), investment was lower. Existing equipment had high maturity with 100% domestication rate.

##### 5.2.1.2 Process flow

A simplified process of “raw material pretreatment → inoculation → solid-state fermentation → drying → packaging” was established. Critical control points (CCPs) included: raw material sterilization (121 °C, 30 min), fermentation temperature and humidity (37 °C ± 1 °C, 55% ± 2%), product moisture (<8%). Process reproducibility was good, with coefficient of variation for enzyme activity <5% in three pilot batches.

##### 5.2.1.3 Quality standards

Enterprise standards were established specifying enzyme activity ≥150 KU/g, moisture ≤8%, and fineness ≥95% (passing 80 mesh). Microbiological safety indicators complied with GB 2760-2014 food additive standards, which defines microbial limits, heavy metals, and safety parameters but not enzyme activity specifications.

#### 5.2.2 Economic benefit prediction

Based on annual production scale of 100 tons:

Investment Estimation: Fixed asset investment of 25 million RMB (workshop 8 million, equipment 12 million, utilities 5 million), working capital 5 million RMB, total investment 30 million RMB.

Revenue Prediction: Annual sales revenue 75 million RMB (selling price 50 RMB/KU × 1.5 million KU), annual production cost 27 million RMB, taxes and management expenses 8 million RMB, annual net profit 40 million RMB. Payback period 2.5 years, internal rate of return (IRR) 28%, higher than industry average for enzyme manufacturing sector (15%–20%) (Anastas and Warner, 1998).

Sensitivity Analysis: With 20% increase in raw material prices, IRR decreased to 24%; with 10% decrease in product price, IRR was 22%; at 70% capacity utilization, IRR was 18%. Project showed strong risk resistance.

### 5.3 Environmental benefit analysis

#### 5.3.1 Resource utilization

Annual consumption of 2,400 tons wheat bran and 1,200 tons soybean meal, converting low-value agricultural byproducts into high-value enzyme preparations, aligning with circular economy principles. Fermentation residue of 3,000 tons/year could be made into organic fertilizer, generating additional revenue of 1.5 million RMB (Singh et al., 2016).

Carbon Emission Reduction: Solid-state fermentation reduced energy consumption by 40% compared to liquid fermentation, equivalent to annual CO_2_ emission reduction of 580 tons. Reduced wastewater discharge by 72,000 tons/year, COD emission reduction of 720 tons/year.

Social Benefits: Project could directly create 50 job positions, drive development of upstream agricultural processing industries, and promote farmer income increase. Low-cost enzyme preparations reduced prices of low-lactose dairy products, benefiting the large lactose-intolerant population, with significant social benefits.

Green Process Indicators: E-factor (waste/product): SSF 0.3, SmF 2.1; Atom economy: SSF 78%, SmF 35%. Solid-state fermentation process complied with green chemistry principles (De Vos et al., 2009), with significantly superior environmental friendliness compared to traditional processes.

## 6 Discussion

### 6.1 Strain screening and solid-state fermentation process innovation

*Lactobacillus plantarum* LP-15 screened from Tibetan fermented yak milk demonstrated significant enzyme production advantages. Its β-galactosidase activity (186.3 U/g after optimization) was significantly higher than reported *L. plantarum* WCFS1 (32.5 U/g) (Delgado-Fernandez et al., 2020) and *L. acidophilus* ATCC 4356 (35.6 U/g) (Huang et al., 2025). This advantage may stem from metabolic adaptive evolution promoted by special selection pressure in high-altitude environments. LP-15 did not produce biogenic amines, showed no hemolytic activity, antibiotic sensitivity met EFSA standards (Binda et al., 2020), demonstrated good food safety, and high genetic stability (enzyme activity variation <3% after 10 successive passages).

The innovation in solid-state fermentation process successfully resolved the compatibility issue between lactic acid bacteria’s high nutritional requirements and SSF technology (Chilakamarry et al., 2022; Kumar et al., 2021). The substrate formula (wheat bran: soybean meal: whey powder = 6:3:1) achieved synergistic effects of carbon-nitrogen source optimization and lactose induction, with 55% moisture content balancing mass transfer and oxygen transfer (Mattedi et al., 2023; Yafetto, 2022). Response surface optimization increased enzyme activity 4.17-fold, breaking the traditional perception that “SSF yield is lower than SmF” (Liu et al., 2025; Menhart et al., 2020). This process reduced production costs by 35.7%, providing an economically feasible technical solution for large-scale production of low-lactose dairy products.

The higher β-galactosidase activity observed in strains from high-altitude regions (Tibet, Xinjiang) may reflect evolutionary adaptations to extreme environmental conditions. High-altitude environments are characterized by lower temperatures, reduced oxygen availability, and increased UV radiation exposure. These stressors may have selected for strains with enhanced metabolic flexibility and stress tolerance mechanisms. Specifically, cold adaptation may have promoted the evolution of enzymes with greater catalytic efficiency to compensate for reduced reaction rates at lower temperatures. Additionally, the unique milk microbiota in traditional fermented yak milk and kumiss may have created distinct selective pressures favoring β-galactosidase production as a competitive advantage for lactose utilization. This geographic pattern suggests that extreme environments may represent valuable sources for isolating industrial strains with superior enzyme production capabilities. However, detailed genomic and transcriptomic studies would be needed to elucidate the specific molecular mechanisms underlying these phenotypic differences.

### 6.2 Comparison of specific activity and process efficiency

The optimization of SSF conditions resulted in a 4.17-fold increase in volumetric activity (from 44.7 to 186.3 U/g substrate), accompanied by a 2.34-fold increase in protein concentration (from 6.5 to 15.2 mg/g) and a 1.78-fold increase in specific activity (from 6.88 to 12.26 U/mg protein) (Table 2). The greater improvement in volumetric activity compared to specific activity indicates that process optimization primarily enhanced total protein synthesis and enzyme production capacity while also moderately improving intrinsic catalytic properties. This is advantageous for industrial application, as higher total enzyme yield per unit substrate directly reduces production costs.

The specific activity of LP-15 crude enzyme (12.26 U/mg) is competitive with other *Lactobacillus* strains cultivated via submerged fermentation (8.2–18.3 U/mg for crude preparations, Supplementary Table S3). Combined with its lower Km value (2.8 mM compared to 3.2–5.2 mM for most LAB β-galactosidases), this demonstrates that SSF using agricultural byproducts can produce enzymes with comparable catalytic activity while offering significant advantages in production costs (35.7% reduction) and environmental impact (94% water reduction, 62.4% CO_2_ reduction).

The optimal substrate ratio (6:3:1) can be explained by complementary nutritional contributions: wheat bran (60%) provides fermentable carbohydrates and porous structure for aeration; soybean meal (30%) supplies protein as nitrogen source; whey powder (10%) provides lactose as specific inducer for β-galactosidase expression. This balanced composition (C/N ratio ∼15:1) matches optimal growth requirements of L. plantarum while enabling effective enzyme induction.

### 6.3 Enzymatic properties and product application advantages

The enzyme produced by LP-15 showed properties highly compatible with dairy processing. The pH optimum of 6.5 was close to milk pH (6.6–6.8), enabling efficient hydrolysis without pH adjustment; good stability at 40 °C (maintaining 85% activity for 4 h) suited low-temperature pasteurization processes (Király et al., 2021); Ca^2+^ activation (125%) was naturally enhanced in calcium-rich milk; low Km value (2.8 mM) ensured high catalytic efficiency (Yan et al., 2021). These properties enabled achievement of low-lactose standard (<0.1%) in 4 h and lactose-free standard (<0.01%) in 6 h, with hydrolysis efficiency comparable to or better than superior to commercial enzyme preparations (Costa et al., 2024; Schulz and Rizvi, 2023).

Product market positioning was clear, targeting China’s 85%–95% lactose-intolerant population (Jiao et al., 2025; Yang et al., 2023), employing a tiered strategy to meet needs of patients with different severity levels (Facioni et al., 2020). Compared to imported products, it had three major advantages: 20%–30% cost reduction, superior sensory quality (8.4 vs. 7.9 points) (Harwood and Drake, 2020), and autonomous supply chain control. With 96.7% of lactose-intolerant individuals experiencing no discomfort after consumption and 93.3% expressing willingness to purchase, the product showed excellent market prospects (Portnoy and Barbano, 2021; Zheng et al., 2023; Dantas et al., 2021).

### 6.4 Research limitations and development directions

Several limitations should be acknowledged in interpreting these findings:

Scale-up Considerations: This study was conducted at laboratory scale (10 g substrate per flask). Industrial-scale operations (>100 kg batches) may face heat and mass transfer challenges due to increased substrate bed depth, potentially affecting temperature distribution, oxygen availability, and moisture uniformity. Pilot-scale validation (50–500 kg) is essential before commercial implementation to optimize parameters for larger reactors.

Enzyme Characterization: While volumetric activity (U/g substrate) and specific activity (U/mg protein) were reported based on crude enzyme extracts, further purification would enable more detailed biochemical characterization and direct comparison with commercial purified enzymes. The crude preparation contains other proteins that may influence performance in specific applications (Duan et al., 2022; Costa et al., 2024).

Economic and Substrate Considerations: The techno-economic analysis is based on June 2024 market prices in eastern China and laboratory-scale yields. Industrial economics will depend on capital investment, local raw material costs, and scale-up efficiency. Additionally, agricultural byproducts exhibit natural compositional variability due to crop varieties and growing conditions, requiring robust quality control protocols.

Application Validation: This study demonstrated efficacy in milk lactose reduction. Performance in other applications (whey processing, pharmaceutical synthesis) requires separate validation, as different matrices may present distinct pH, temperature, or substrate accessibility challenges.

Despite these limitations, the demonstrated cost reduction (35.7%), improved specific activity (1.78-fold), and favorable environmental profile provide a strong foundation for further development toward commercial viability.

## 7 Conclusion

This study effectively addressed the challenges of high-cost imported β-galactosidase in China’s low-lactose dairy market. We isolated *Lactobacillus plantarum* LP-15 from Tibetan fermented yak milk, showing initial enzyme activity of 44.7 U/g. Through Box-Behnken optimization of solid-state fermentation using agricultural byproducts (wheat bran: soybean meal: whey powder = 6:3:1) at 37 °C, pH 6.5, and 55% moisture content, enzyme activity reached 186.3 ± 2.1 U/g—a 4.17-fold improvement. The enzyme exhibited highest activity at pH 6.5 °C and 42 °C, with Km of 2.8 mM and catalytic efficiency of 1.2 × 10^5^ M^-1^s^-1^. Under optimized conditions (2.0 U/mL, 40 °C), milk lactose was reduced from 4.8% to 0.09% within 4 h (low-lactose standard) and below 0.01% within 6 h (lactose-free standard), with 96.7% of lactose-intolerant consumers experiencing no discomfort.

Techno-economic analysis revealed that solid-state fermentation reduced production costs by 35.7% compared to liquid fermentation, with an internal rate of return of 28% and payback period of 2.5 years. The process decreased water usage by 94% and CO_2_ emissions by 62.4%, while converting 3,600 tons of agricultural byproducts annually into high-value enzyme preparations. This study provides China’s low-lactose dairy industry with a cost-effective, environmentally sustainable technology with independent intellectual property rights, breaking foreign monopoly and offering significant benefits for the country’s 85%–95% lactose-intolerant population while advancing circular economy principles in agricultural waste utilization.

## Data Availability

The original contributions presented in the study are included in the article/Supplementary Material, further inquiries can be directed to the corresponding author.
