# Supplementary material for: Optimization of solid-state fermentation conditions for high β-galactosidase-producing lactic acid bacteria and its application in low-lactose dairy products
Source: Front Bioeng Biotechnol. 2025 Dec 11;13:1708601. doi: 10.3389/fbioe.2025.1708601 (PMC12739383; doi:10.3389/fbioe.2025.1708601)
Supplement: Supplementary file 1 [file Table1.docx]

**Supplementary Material**

**S1. Sensory Evaluation Methodology**

Ethical Considerations

The sensory evaluation was conducted in accordance with the Declaration of Helsinki and ethical principles for research involving human subjects. All participants provided written informed consent before participation, which included information about the study purpose, procedures, potential risks and benefits, and their right to withdraw at any time without penalty. Personal data and responses were anonymized and stored confidentially in compliance with data protection regulations. Participants were assigned random identification codes, and no personally identifiable information was linked to sensory data.

Participant Recruitment

A total of 100 consumers were recruited through public advertisement and screening questionnaire. Inclusion criteria: (1) aged 18-60 years; (2) regular dairy product consumers (≥3 times/week); (3) no food allergies or sensitivities; (4) non-smokers; (5) no recent use (within 2 weeks) of medications affecting taste or smell perception; (6) willing to provide informed consent.

The panel consisted of 60 self-reported lactose-intolerant individuals (confirmed by previous medical diagnosis or positive hydrogen breath test results) and 40 lactose-tolerant individuals (control group). Participants received compensation (50 RMB) for their time and were informed of their right to withdraw at any time without penalty.

Sample Preparation

Low-lactose whole milk samples were prepared using LP-15 β-galactosidase under optimal hydrolysis conditions (2.0 U/mL, 40°C, 4 h). Commercially available imported low-lactose milk was purchased as control sample. All samples were stored at 4°C and equilibrated to 6-8°C before serving.

Samples (50 mL each) were served in odorless white plastic cups (100 mL capacity) labeled with random three-digit codes. Sample presentation order was randomized using a balanced Latin square design.

Evaluation Environment

Sensory evaluation was conducted in individual sensory booths meeting ISO 8589:2007 requirements. Environmental conditions: temperature 20±2°C, relative humidity 60±10%, white LED lighting ≥1000 lux, odor-free and noise-controlled (<50 dB). Each booth was equipped with a computer for data entry and provided with unsalted crackers and room temperature water for palate cleansing.

Evaluation Procedure

Participants were briefed on the evaluation procedure and rating scales. The evaluation consisted of:

1. Palate Preparation: Mouth rinse with water, 2-minute wait

2. Sample Evaluation: Five sensory attributes rated using 9-point hedonic scale:

- Color (1 = extremely dislike, 9 = extremely like)

- Aroma (1 = extremely unpleasant, 9 = extremely pleasant)

- Taste (1 = extremely dislike, 9 = extremely like)

- Sweetness (1 = extremely inadequate, 9 = extremely appropriate)

- Overall acceptability (1 = extremely unacceptable, 9 = extremely acceptable)

3. Inter-sample Cleansing: Crackers and water, 30-second wait

4. Purchase Intention: Yes/No for each sample

5. Post-consumption Monitoring: Gastrointestinal discomfort assessed 2 hours after consumption (5-point scale: 1 = no discomfort, 5 = severe discomfort)

Data Collection

Data were collected using computerized data entry system (Compusense Cloud, Compusense Inc., Canada). Participants entered ratings directly, with automatic recording of timestamp and sample codes.

Statistical Analysis

Data were analyzed using SPSS 22.0. Normality was assessed using Shapiro-Wilk test. For normally distributed data, one-way ANOVA with Tukey's HSD post-hoc test was performed. For non-normally distributed data, Kruskal-Wallis H test was used. Statistical significance: P < 0.05.

Spider plots were generated using Origin 9.0. Purchase intention and gastrointestinal comfort data were analyzed using chi-square test or Fisher's exact test.

Quality Control

Quality measures: (1) duplicate samples for consistency assessment; (2) triangle tests during recruitment; (3) outlier screening using boxplot analysis; (4) exclusion of inconsistent panelists (>30% deviation from group mean).

**S2. Physiological and Biochemical Characterization Methods**

Morphological Characterization

Gram staining was performed using a Gram staining kit (Solarbio, Beijing, China) following the manufacturer's instructions. Fresh bacterial colonies were heat-fixed on glass slides, stained sequentially with crystal violet, iodine solution, decolorized with 95% ethanol, and counterstained with safranin. Cell morphology was observed under a light microscope (Olympus BX53, Japan) at 1000× magnification.

For scanning electron microscopy (SEM), bacterial cells were fixed with 2.5% glutaraldehyde in phosphate buffer (pH 7.2) for 2 h at 4°C, washed three times with the same buffer, and post-fixed with 1% osmium tetroxide for 1 h. Samples were dehydrated through a graded ethanol series (30%, 50%, 70%, 85%, 95%, 100%), critical-point dried, mounted on aluminum stubs, and sputter-coated with gold. Cell morphology was examined using a scanning electron microscope (JSM-6510LV, JEOL, Japan) at 15 kV acceleration voltage.

Biochemical Tests

Catalase Test: Fresh bacterial colonies were transferred to a clean glass slide, and 3% (v/v) hydrogen peroxide was added. Immediate bubble formation indicated positive catalase activity.

Anaerobic Growth: Strains were cultured in MRS broth under anaerobic conditions using AnaeroPack sachets (Mitsubishi Gas Chemical, Japan) in anaerobic jars at 37°C for 48 h. Growth was assessed by measuring optical density at 600 nm (OD_600_).

Gelatin Hydrolysis: Strains were stabbed into gelatin agar medium (12% gelatin, nutrient broth base) and incubated at 37°C for 7 days. After incubation, plates were refrigerated at 4°C for 4 h. Liquefaction of gelatin indicated positive hydrolysis.

Nitrate Reduction: Strains were cultured in nitrate broth (nutrient broth containing 0.1% potassium nitrate) at 37°C for 48 h. Nitrite formation was detected by adding Griess reagent (sulfanilic acid and α-naphthylamine in acetic acid). Development of red color indicated positive nitrate reduction.

Carbohydrate Fermentation

Carbohydrate fermentation tests were conducted in phenol red broth base containing 1% (w/v) of individual carbon sources: glucose, fructose, galactose, lactose, maltose, sucrose, mannitol, sorbitol, cellobiose, salicin, trehalose, xylose, arabinose, rhamnose, and raffinose. Bromocresol purple (0.004% w/v) was used as pH indicator. Each carbohydrate was filter-sterilized (0.22 μm) and added aseptically to autoclaved basal medium. Cultures were incubated at 37°C for 48 h. Color change from purple to yellow (pH < 5.2) indicated acid production and positive fermentation. All tests were performed in triplicate.

Arginine Hydrolysis

Arginine broth (1% tryptone, 0.5% yeast extract, 0.5% $K_2HPO_4$, 0.2% glucose, 0.3% L-arginine, 0.002% bromocresol purple, pH 6.0) was inoculated and incubated at 37°C for 7 days. After incubation, Nessler's reagent (potassium mercuric iodide solution) was added. Development of orange-brown color indicated ammonia production and positive arginine hydrolysis.

Citrate Utilization

Simmons citrate agar slants (sodium citrate 0.2%, ammonium dihydrogen phosphate 0.1%, K_2_HPO_4_ 0.1%, NaCl 0.5%, MgSO_4_ 0.02%, bromothymol blue 0.008%, agar 1.5%) were inoculated and incubated at 37°C for 7 days. Color change from green to blue indicated citrate utilization.

Growth Characteristics

pH Tolerance: MRS broth was adjusted to pH 4.5, 5.0, 5.5, 6.0, 6.5, 7.0, 7.5, and 8.0 using 1 M HCl or 1 M NaOH before autoclaving. Strains were inoculated (2% v/v) and incubated at 37°C. Growth was measured by OD_600_ at 0, 12, 24, 36, and 48 h. Growth curves were plotted, and optimal pH was determined as the pH yielding maximum growth rate.

Temperature Tolerance: Cultures were incubated in MRS broth at 10°C, 15°C, 20°C, 25°C, 30°C, 37°C, 40°C, 43°C, and 45°C for 48 h. Growth was assessed by measuring OD_600_. Temperatures supporting OD_600_ > 0.3 after 48 h were considered permissive for growth. Optimal temperature was determined as the temperature yielding highest growth rate in the exponential phase.

Salt Tolerance: MRS broth was supplemented with 0%, 2%, 4%, 6.5%, 8%, and 10% (w/v) NaCl. Strains were inoculated and incubated at 37°C for 48 h. Growth was assessed by measuring OD_600_. Maximum tolerated NaCl concentration was defined as the highest concentration supporting visible growth (OD_600_ > 0.3).

Statistical Analysis

All experiments were performed in triplicate. Results are expressed as mean ± standard deviation. Growth characteristics (pH, temperature, salt tolerance) were analyzed using GraphPad Prism 8.0 software.

**S3. Safety Evaluation Methods**

Biogenic Amine Detection

Biogenic amines were detected by high-performance liquid chromatography (HPLC) following the method of Benkerroum (2016). Strain LP-15 was cultured in MRS broth containing precursor amino acids (tyrosine 3 g/L, histidine 2 g/L, ornithine 2 g/L, lysine 2 g/L, phenylalanine 2 g/L, tryptophan 1 g/L) at 37°C for 48 h. Culture supernatants were collected by centrifugation (10,000×g, 10 min, 4°C) and filtered (0.22 μm).

For derivatization, 1 mL of sample was mixed with 500 μL of saturated sodium bicarbonate solution and 2 mL of dansyl chloride solution (10 mg/mL in acetone). The mixture was incubated at 40°C for 45 min in darkness, then 100 μL of 25% ammonia solution was added to stop the reaction. After incubation at room temperature for 30 min, samples were extracted with 2 mL of diethyl ether, and the organic phase was evaporated under nitrogen. The residue was dissolved in 1 mL of acetonitrile and filtered (0.22 μm).

HPLC analysis was performed using a C18 column (250 × 4.6 mm, 5 μm, Agilent) at 30°C. Mobile phase A: 0.1 M ammonium acetate (pH 6.5); Mobile phase B: acetonitrile. Gradient elution: 0-5 min 50% B, 5-30 min 50-95% B, 30-35 min 95% B, 35-40 min 95-50% B. Flow rate: 1.0 mL/min. Detection: UV absorbance at 254 nm. Biogenic amine standards (histamine, tyramine, putrescine, cadaverine, tryptamine, phenylethylamine) were purchased from Sigma-Aldrich. Detection limit: 5 mg/L.

Hemolytic Activity Test

Hemolytic activity was assessed on blood agar plates. Fresh overnight cultures were streaked on Columbia blood agar base (Oxoid) supplemented with 5% (v/v) defibrinated sheep blood. Plates were incubated at 37°C for 48 h under anaerobic conditions. Staphylococcus aureus ATCC 25923 was used as positive control (β-hemolysis), and Lactobacillus rhamnosus GG as negative control (γ-hemolysis). Hemolytic patterns were classified as: α-hemolysis (partial, greenish zone), β-hemolysis (complete, clear zone), or γ-hemolysis (no hemolysis).

Antibiotic Susceptibility Testing

Minimum inhibitory concentrations (MICs) were determined using the broth microdilution method according to ISO 10932:2010 and EFSA guidelines. Twelve antibiotics were tested: ampicillin, penicillin G, erythromycin, tetracycline, chloramphenicol, clindamycin, gentamicin, kanamycin, streptomycin, vancomycin, ciprofloxacin, and trimethoprim.

Bacterial suspensions were prepared to 0.5×10^8^ CFU/mL in LSM broth (90% Iso-Sensitest broth + 10% MRS broth). Antibiotic stock solutions were prepared in appropriate solvents and serially diluted in 96-well microplates to achieve final concentrations ranging from 0.125 to 512 μg/mL. Each well contained 100 μL of antibiotic solution and 100 μL of bacterial suspension (final concentration: 2.5×10^7^ CFU/mL). Plates were incubated at 37°C for 48 h under anaerobic conditions. MIC was defined as the lowest antibiotic concentration inhibiting visible growth. All tests were performed in triplicate.

EFSA microbiological breakpoints for Lactobacillus spp. were used as reference values.

Detection of Antibiotic Resistance Genes

Genomic DNA was extracted using a Bacterial Genomic DNA Extraction Kit (Tiangen Biotech) following the manufacturer's protocol. PCR amplification was performed to detect transferable antibiotic resistance genes (ermB, ermC, tetM, tetS, vanA, vanB).

PCR primers:

- ermB: Forward 5'-GAAAAGGTACTCAACCAAATA-3', Reverse 5'-AGTAACGGTACTTAAATTGTTTAC-3' (639 bp)

- ermC: Forward 5'-TCAAAACATAATATAGATAAA-3', Reverse 5'-GCTAATATTGTTTAAATCGTCAAT-3' (572 bp)

- tetM: Forward 5'-GTGGACAAAGGTACAACGAG-3', Reverse 5'-CGGTAAAGTTCGTCACACAC-3' (406 bp)

- tetS: Forward 5'-GAAAGCTTACTATACAGTAGC-3', Reverse 5'-AGGAGTATCTACAATATTTAC-3' (169 bp)

PCR conditions: initial denaturation at 95°C for 5 min; 35 cycles of 95°C for 30 s, 55°C for 30 s, 72°C for 1 min; final extension at 72°C for 10 min. PCR products were analyzed by 1.5% agarose gel electrophoresis.

Cell Adhesion Assay

Caco-2 cells (human colon adenocarcinoma cell line, ATCC HTB-37) were cultured in DMEM medium supplemented with 10% FBS, 1% non-essential amino acids, and 1% antibiotics at 37°C in 5% CO_2_ atmosphere. Cells were seeded in 24-well plates (2×10^5^ cells/well) and grown to 90% confluence.

Before adhesion assay, Caco-2 monolayers were washed three times with sterile PBS. Bacterial suspensions were prepared in antibiotic-free DMEM to 1×10^8^ CFU/mL. 1 mL of bacterial suspension was added to each well (MOI = 100:1) and incubated at 37°C for 2 h in 5% CO_2_. Non-adhered bacteria were removed by washing three times with sterile PBS. Adhered bacteria and cells were lysed with 0.1% Triton X-100 for 10 min. Serial dilutions were plated on MRS agar and incubated at 37°C for 48 h to enumerate adhered bacteria.

Adhesion rate (%) = (CFU of adhered bacteria / CFU of initial bacteria) × 100

L. plantarum 299v (DSM 6595) and L. plantarum ATCC 8014 were used as positive and reference controls, respectively.

Simulated Gastrointestinal Tolerance

Artificial Gastric Juice (AGJ): 0.2% (w/v) NaCl, 0.35% (w/v) pepsin (≥2500 U/mg, Sigma-Aldrich), adjusted to pH 2.5 with 1 M HCl, filter-sterilized (0.22 μm).

Artificial Intestinal Juice (AIJ): 0.68% (w/v) KH_2_PO_4_, 0.1% (w/v) trypsin (≥2500 U/mg, Sigma-Aldrich), adjusted to pH 8.0 with 1 M NaOH, filter-sterilized (0.22 μm).

Overnight cultures were harvested by centrifugation (5,000×g, 10 min, 4°C), washed twice with sterile PBS, and resuspended to 10^9^ CFU/mL. For gastric tolerance, 1 mL of bacterial suspension was mixed with 9 mL of AGJ and incubated at 37°C with shaking (150 rpm). Samples were taken at 0, 1, 2, and 3 h. For intestinal tolerance, bacterial pellets after gastric treatment were washed with PBS and resuspended in AIJ, incubated at 37°C for 4 h. Viable counts were determined by serial dilution plating on MRS agar.

Survival rate (%) = (log CFU at time t / log CFU at time 0) × 100

Bile Salt Tolerance

Bile salt tolerance was evaluated by adding oxgall (Sigma-Aldrich) to MRS broth at final concentrations of 0%, 0.1%, 0.3%, 0.5%, and 1.0% (w/v). Overnight cultures were inoculated (2% v/v) and incubated at 37°C. Growth was monitored by measuring OD_600_ at 0, 2, 4, 6, and 8 h. Viable counts were determined at 4 h by serial dilution plating.

Bile tolerance was expressed as log reduction: Δlog CFU = log CFU (0% bile) - log CFU (0.3% bile)

Statistical Analysis

All experiments were performed in triplicate. Data are expressed as mean ± standard deviation. Statistical differences were analyzed using one-way ANOVA followed by Tukey's post-hoc test. P < 0.05 was considered statistically significant. GraphPad Prism 8.0 was used for data analysis.

**Table S1.** Physiological and Biochemical Characteristics of Lactobacillus plantarum LP-15

| **Characteristic** | **Test/Substrate** | **Result** |
| --- | --- | --- |
| **MORPHOLOGICAL CHARACTERISTICS** | | |
| Colony morphology | MRS agar | Circular, smooth, milky white |
| Colony size (mm) | MRS agar, 48h | 1.5-2.0 |
| Cell morphology | Gram staining | Short rod to rod-shaped |
| Cell size (μm) | Microscopy | (0.6-0.8) × (2.0-3.5) |
| Cell arrangement | Microscopy | Single, pairs, short chains |
| Gram staining | Crystal violet | Positive (+) |
| Spore formation | Microscopy | Negative (-) |
| Flagella | Microscopy | Negative (-) |
| **PHYSIOLOGICAL CHARACTERISTICS** | | |
| Oxygen requirement | Aerobic/anaerobic | Facultatively anaerobic |
| Catalase test | H₂O₂ (3%) | Negative (-) |
| Gelatin hydrolysis | Gelatin agar | Negative (-) |
| Nitrate reduction | Nitrate broth | Negative (-) |
| Arginine hydrolysis | Arginine broth | Negative (-) |
| Citrate utilization | Simmons citrate agar | Positive (+) |
| **CARBOHYDRATE FERMENTATION** | | |
| Glucose | Phenol red broth | Positive (+) |
| Fructose | Phenol red broth | Positive (+) |
| Galactose | Phenol red broth | Positive (+) |
| Lactose | Phenol red broth | Positive (+) |
| Maltose | Phenol red broth | Positive (+) |
| Sucrose | Phenol red broth | Positive (+) |
| Mannitol | Phenol red broth | Positive (+) |
| Sorbitol | Phenol red broth | Positive (+) |
| Cellobiose | Phenol red broth | Positive (+) |
| Salicin | Phenol red broth | Positive (+) |
| Trehalose | Phenol red broth | Positive (+) |
| Xylose | Phenol red broth | Negative (-) |
| Arabinose | Phenol red broth | Negative (-) |
| Rhamnose | Phenol red broth | Negative (-) |
| Raffinose | Phenol red broth | Negative (-) |
| **GROWTH CHARACTERISTICS** | | |
| pH tolerance range | MRS broth | pH 4.5-8.0 |
| Optimal pH | MRS broth | pH 6.0-6.5 |
| Growth at pH 4.5 | MRS broth, 48h | Weak (+) |
| Growth at pH 5.0 | MRS broth, 48h | Good (++) |
| Growth at pH 6.0-7.0 | MRS broth, 48h | Excellent (+++) |
| Growth at pH 8.0 | MRS broth, 48h | Moderate (++) |
| Temperature tolerance range | MRS broth | 15-45°C |
| Optimal temperature | MRS broth | 37°C |
| Growth at 10°C | MRS broth, 7 days | Slow (+) |
| Growth at 15°C | MRS broth, 48h | Weak (+) |
| Growth at 37°C | MRS broth, 48h | Excellent (+++) |
| Growth at 43°C | MRS broth, 48h | Good (++) |
| Growth at 45°C | MRS broth, 48h | Negative (-) |
| Salt (NaCl) tolerance | MRS broth + NaCl | Up to 6.5% (w/v) |
| Growth at 2% NaCl | MRS broth, 48h | Excellent (+++) |
| Growth at 4% NaCl | MRS broth, 48h | Good (++) |
| Growth at 6.5% NaCl | MRS broth, 48h | Weak (+) |
| Growth at 8% NaCl | MRS broth, 48h | Negative (-) |

*Note: All tests were performed in triplicate. Growth intensity: (-) no growth, (+) weak growth (OD_600_ < 0.3), (++) moderate growth (OD_600_ 0.3-0.8), (+++) strong growth (OD_600_ > 0.8). These characteristics are consistent with the standard description of Lactobacillus plantarum according to Bergey's Manual of Systematic Bacteriology.*

**Table S2.** Complete Analysis of Variance (ANOVA) and Regression Coefficients for Box-Behnken Response Surface Model

**Part A:** Analysis of Variance (ANOVA)

| **Source** | **DF** | **Sum of Squares** | **Mean Square** | **F Value** | **p-value** |
| --- | --- | --- | --- | --- | --- |
| **Model** | 9 | 3421.67 | 380.19 | 61.85 | <0.0001*** |
| X_1_ - Temperature | 1 | 538.24 | 538.24 | 87.56 | <0.0001*** |
| X_2_ - pH | 1 | 359.46 | 359.46 | 58.48 | 0.0001*** |
| X_3_ - Moisture | 1 | 224.68 | 224.68 | 36.56 | 0.0005*** |
| X_1_X_2_ | 1 | 282.24 | 282.24 | 45.92 | 0.0003*** |
| X_1_X_3_ | 1 | 153.76 | 153.76 | 25.02 | 0.0016** |
| X_2_X_3_ | 1 | 125.44 | 125.44 | 20.41 | 0.0028** |
| X_1_^2^ | 1 | 600.43 | 600.43 | 97.68 | <0.0001*** |
| X_2_^2^ | 1 | 375.28 | 375.28 | 61.06 | <0.0001*** |
| X_3_^2^ | 1 | 225.52 | 225.52 | 36.70 | 0.0005*** |
| **Residual** | 7 | 43.03 | 6.15 |  |  |
| Lack of Fit | 3 | 18.94 | 6.31 | 1.05 | 0.4523 |
| Pure Error | 4 | 24.09 | 6.02 |  |  |
| **Cor Total** | 16 | 3464.70 |  |  |  |

**Part B:** Regression Coefficients

| **Term** | **Coefficient** | **Std Error** | **t Value** | **p-value** |
| --- | --- | --- | --- | --- |
| Intercept (β_0_) | 186.30 | 1.43 | 130.21 | <0.0001*** |
| X_1_ (β_1_) | 8.20 | 0.88 | 9.36 | <0.0001*** |
| X_2_ (β_2_) | 6.70 | 0.88 | 7.65 | 0.0001*** |
| X_3_ (β_3_) | 5.30 | 0.88 | 6.05 | 0.0005*** |
| X_1_X_2_ (β_12_) | 4.20 | 0.62 | 6.78 | 0.0003*** |
| X_1_X_3_ (β_13_) | 3.10 | 0.62 | 5.00 | 0.0016** |
| X_2_X_3_ (β_23_) | 2.80 | 0.62 | 4.52 | 0.0028** |
| X_1_^2^ (β_11_) | -12.40 | 1.25 | -9.88 | <0.0001*** |
| X_2_^2^ (β_22_) | -9.80 | 1.25 | -7.81 | <0.0001*** |
| X_3_^2^ (β_33_) | -7.60 | 1.25 | -6.06 | 0.0005*** |

**Part C:** Model Quality Statistics

| **Statistic** | **Value** |
| --- | --- |
| R² | 0.9876 |
| Adjusted R² | 0.9716 |
| Predicted R² | 0.9432 |
| Adequate Precision | 24.58 |
| RMSE (Root Mean Square Error) | 2.48 U/g |
| Mean | 165.83 U/g |
| Coefficient of Variation (C.V. %) | 2.34 |
| Lack-of-fit p-value | 0.4523 (Not Significant) |

*Notes: Significance levels: *p < 0.05; **p < 0.01; ***p < 0.001; X₁ = Temperature (°C); X₂ = pH; X₃ = Moisture content (%); DF = Degrees of Freedom; The lack-of-fit test result (p = 0.4523 > 0.05) confirms that the model adequately describes the experimental data with no systematic lack of fit; Adequate Precision measures signal-to-noise ratio; values >4 indicate adequate model discrimination. The value of 24.58 demonstrates excellent model discrimination capability; High R² (0.9876) and close agreement between Adjusted R² (0.9716) and Predicted R² (0.9432) indicate good model fit and prediction capability.*

**Table S3.** Comparison of Specific Activity with Literature-Reported LAB β-Galactosidases

| **Strain** | **Production Method** | **Specific Activity (U/mg)** | **Km (mM)** | **Reference** |
| --- | --- | --- | --- | --- |
| L. plantarum LP-15 (this study) | SSF (optimized) | 12.26 | 2.8 | This study |
| L. plantarum WCFS1 | SmF | 8.2 | 4.5 | Delgado et al., 2020 |
| L. rhamnosus | SmF | 12.4 | 3.8 | Huang et al., 2025 |
| L. fermentum | SmF | 15.6 | 5.2 | Mahadevaiah et al., 2020 |
| L. helveticus | SmF | 18.3 | 3.2 | Isolation and characterization of a β-galactosidase from Lactobacillus helveticus for industrial processing (2024) |
| Commercial Lactozym (K. lactis) | Purified enzyme | 45-65 | 2.1 | Novozymes data |
| Commercial Maxilact (K. fragilis) | Purified enzyme | 38-52 | 2.8 | DSM data |

*Note: SSF = solid-state fermentation; SmF = submerged (liquid) fermentation. Specific activity values for LAB strains refer to crude or partially purified enzyme preparations. Commercial enzymes are highly purified preparations. The LP-15 enzyme demonstrates competitive specific activity among LAB-derived β-galactosidases while maintaining excellent substrate affinity (lower Km) compared to most reported strains. The specific activity values are based on Bradford assay quantification of crude enzyme extracts, representing practical enzyme preparations from fermentation processes.*
